# JAK/STAT inhibitor therapy partially rescues the lipodystrophic autoimmune phenotype in *Clec16a* KO mice

**DOI:** 10.1038/s41598-021-86493-8

**Published:** 2021-04-01

**Authors:** Rahul Pandey, Marina Bakay, Bryan P. Strenkowski, Heather S. Hain, Hakon Hakonarson

**Affiliations:** 1 The Center for Applied Genomics, The Children’s Hospital of Philadelphia, Philadelphia, PA 19104 USA; 2 Department of Pediatrics, The Perelman School of Medicine, University of Pennsylvania, Philadelphia, PA 19104 USA

**Keywords:** Immunology, Diseases

## Abstract

*CLEC16A* is implicated in multiple autoimmune diseases. We generated an inducible whole-body knockout (KO), *Clec16a*^*ΔUBC*^ mice to address the role of *CLEC16A* loss of function. KO mice exhibited loss of adipose tissue and severe weight loss in response to defective autophagic flux and exaggerated endoplasmic reticulum (ER) stress and robust cytokine storm. KO mice were glucose tolerant and displayed a state of systemic inflammation with elevated antibody levels, including IgM, IgA, Ig2b and IgG3, significantly reduced circulating insulin levels in the presence of normal food consumption. Metabolic analysis revealed disturbances in the lipid profile, white adipose decreasing concomitantly with enhanced inflammatory response, and energy wasting. Mechanistically, endoplasmic reticulum (ER) stress triggers excessive hormone sensitive lipases (HSL) mediated lipolysis which contributes to adipose inflammation via activation of JAK-STAT, stress kinases (ERK1/2, P38, JNK), and release of multiple proinflammatory mediators. Treatment with a JAK-STAT inhibitor (tofacitinib) partially rescued the inflammatory lipodystrophic phenotype and improved survival of *Clec16a*^*ΔUBC*^ mice by silencing cytokine release and modulating ER stress, lipolysis, mitophagy and autophagy. These results establish a mechanistic link between *CLEC16A*, lipid metabolism and the immune system perturbations. In summary, our *Clec16a*^*ΔUBC*^ mouse model highlights multifaceted roles of *Clec16a* in normal physiology, including a novel target for weight regulation and mutation-induced pathophysiology.

## Introduction

Synergistic combination of genetic predisposition, largely unknown environmental triggers, and immunologic events leads to autoimmune disease onset. *CLEC16A* is a well-established T1DM susceptibility gene which also has been associated with the susceptibility to several other autoimmune diseases, including Type1 diabetes (T1D)^1–5^, multiple sclerosis (MS)^6–8^, primary adrenal insufficiency (PAI)^9^, systemic lupus erythematosus (SLE)^10, 11^, Crohn’s disease (CD)^12^, selective immunoglobulin A deficiency (IgA)^9^, alopecia areata (AA)^13, 14^, juvenile idiopathic arthritis (JIA)^15^, rheumatoid arthritis (RA)^15, 16^, primary biliary cirrhosis (PBC)^17, 18^ and asthma^19–21^. The shared association of *CLEC16A* in these diverse inflammatory and autoimmune diseases suggests that *CLEC16A* could be a critical regulator of autoimmune responses. Mitochondrial dysfunction, persistent ER stress, oxidative stress, inflammation, and altered lipid metabolism, all of which have been attributed to loss of function variants in *CLEC16A*, may be the key mechanisms involved in the pathogenesis of autoimmune diseases. As a consequence, *CLEC16A* has become an attractive candidate for functional studies to explore the pathogenic mechanisms and potential therapeutic options through *CLEC16A* intervention in autoimmune diseases.

In 2007, we first identified the region mapping to KIAA0350^1^ (now called C-type lectin-like domain family 16A (*CLEC16A*)) as a novel T1D susceptibility locus within a 233- kb LD block on chromosome 16p13. *CLEC16A* encodes an E3 ligase that promotes Nrdp1 ubiquitination and is ubiquitously expressed gene with functions in other cell types, including immune cells and neurons^22–25^. Previously, we showed that *CLEC16A* is required for normal glucose stimulated insulin release through its effect on mitophagy using mice with a pancreas specific deletion^23^. Evidence indicates that mitochondria lie at the heart of immunity and are signaling hubs^26–29^. Mitophagy functions as an early protective response, favoring adaptation to stress by removing damaged mitochondria. Maintaining mitochondrial fidelity is therefore important to elicit physiologic signaling responses, homeostasis and immune cell survival. When this process is derailed, dysfunctional mitochondria can contribute to human diseases by damaging proteins and lipids in response to hyperactive inflammatory pathways, including mediators attributed to Janus kinase/signal transducer and activator of transcription (JAK/STAT) signaling^30–32^. Results in our laboratory revealed incomplete mitophagy predisposes *Clec16a*^*ΔUBC*^ mice to a cascade of altered immune signaling functions leading to pathogenic inflammation^33^. We also showed *Clec16a* restrains natural killer (NK) cell function in the YTS NK cell line and *Clec16a*^*ΔUBC*^ mice, indicating a delicate balance of *CLEC16A* is needed for NK cell function and homeostasis, including cytokine release and cytotoxicity^34^.

Previous studies implicate a role for cellular stress response in a wide range of autoimmune diseases^35–38^. ER stress regulates cytokine production through variety of mechanism and contributes to immune responses by enhancing inflammatory signaling and cytokine transcription activation^39^. Cytokines critically mediate host defense against pathogens, but when aberrantly produced, may also drive pathogenic inflammation^40^. Beyond cytokine regulation, the ER/unfolded protein response (UPR) influences autophagy, nutrient mobilization and the cell death^36, 41–43^. Numerous signaling components and pathways interweave the (UPR) with inflammation and initiate autophagy in response to cellular stress thus making ER an effective nidus for promoting inflammation^35, 36, 40, 44^.

Severe mitochondrial dysfunction is known to inhibit the expression of PPARγ^45^, induce cell apoptosis^46^ resulting in lipoatrophy^47^. Therefore, dysfunctional adipose tissue and mitochondria are at the very core of metabolic dysfunction and together can trigger the pathogenic autoimmune response and signaling event in genetically susceptible individuals. Recent studies highlighted the role of *CLEC16A* as critical regulator of mitophagy^23^ and autophagy^48^. Adipose tissue is a complex organ and plays an active role in whole body energy and metabolic homeostasis. Its excess (e.g. obesity) or absence (e.g. lipodystrophy) is associated with severe metabolic disease^49^. Two functionally distinct types of adipose tissue with opposing metabolic properties, white adipose tissue (WAT) and brown adipose tissue (BAT), exist in mammals. WAT is the primary energy reservoir and a major source of metabolic fuel in response to energy demands. WAT further integrates metabolic signals and regulates systemic energy balance by secreting adipokines, including leptin, adiponectin and tumor necrosis factor-alpha (TNF-α)^50^. ER stress is known to activate lipolytic cascade in adipocytes^51–53^. Depletion of WAT through excessive adipocyte lipolysis generates lipid mediators, high systemic levels of acute phase reaction proteins, inflammatory cytokines and triggers inflammation. BAT is specialized for energy expenditure and adaptive thermogenesis in response to cold exposure. Lipolysis and lipophagy are the two major lipid metabolism pathways identified in humans^54^.

We hypothesized that in response to dysregulated autophagy and mitophagy, adipose tissue in *Clec16a*^*ΔUBC*^ mice exhibits deregulated ER homeostasis which activates the lipolytic cascade. We show that *Clec16a*^*ΔUBC*^ mice display non-preferential loss of body fat despite no difference in food intake which is associated with defective autophagic flux in adipose tissue, resulting in ER stress. Downstream ER stress activates lipolytic cascade in adipose tissue which triggers inflammatory response with increased oxidative stress and production of inflammatory mediators, a process that is likely to contribute to the pathogenesis of autoimmune disorders, associated with *CLEC16A*. We additionally demonstrate that a pan-JAK/STAT inhibitor drug had a multifaceted effect and partially rescued the lipodystrophy and inflammatory phenotype by modulating ER stress. Taken together, our inducible *Clec16a*^*ΔUBC*^ mouse model demonstrates loss of function of *CLEC16A* elicits a derailed ER stress induced lipolytic cascade under compromised mitophagy/autophagy conditions involving JAK-STAT/SOCS signaling, a pathological process rescued in part by JAK/STAT inhibitor drugs. Thus, interventions directed at reversing the consequences of loss of function in *CLEC16A* could present an effective therapy for autoimmune and inflammatory disorders.

## Results

### Defective autophagy and ER stress contributes to adipose tissue atrophy in *Clec16a*^ΔUBC^ knockout mice

**Figure 1:**
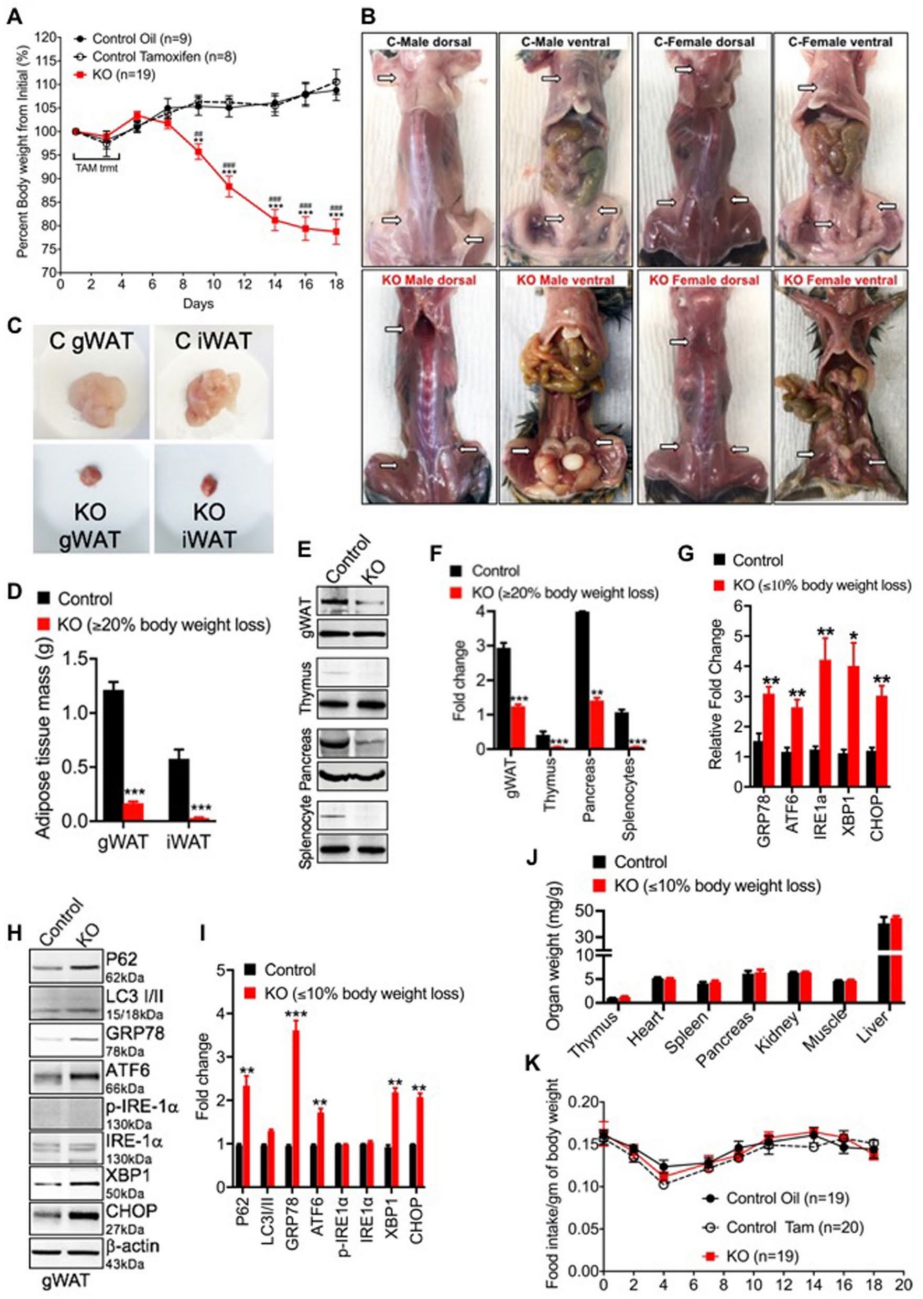
Defective autophagy and ER stress mediated adipose tissue atrophy in *Clec16a*^*ΔUBC*^* mice*. (**A**) Body weight was measured three times/week for control and *Clec16a*^*ΔUBC*^* mice* fed with standard rodent chow. Data are presented as Mean ± SE, n = 9 (oil control), n = 8 (tamoxifen control), n = 19 (KO) mice/group. Two-way ANOVA. Tukey's multiple Comparison test, **p < 0.001, ****p < 0.0001 compared to the control group. (**B**) Representative dorsal and ventral images depict gross morphology and distribution of fat in tamoxifen control (top panel), *Clec16a*^*ΔUBC*^ (KO)**,** bottom panel) male (left) and female (right) mice. (**C**) Representative images of gonadal (gWAT) and inguinal (IWAT) fat pads at sacrifice. (**D**) Graph depicts white adipose tissue (gWAT and IWAT) weights at sacrifice (n = 9 mice/group). (**E**) Representative immunoblot depicts CLEC16A expression in gWAT, thymus, pancreas and splenocyte lysate of tamoxifen control and *Clec16a*^*ΔUBC*^. (**F**) Graph depicts CLEC16A protein expression. Data presented as Mean ± SE of three independent repeats. (**G**) mRNA levels of ER stress markers in tamoxifen control and KO gWAT. (**H**) Representative immunoblot depicts expression of P62, LC3-I/II and ER stress markers in *Clec16a*^*ΔUBC*^ gWAT lysate. Membranes were striped and reprobed for β-actin as a loading control. (**I**) Graph depicts P62, LC3-I/II and ER stress markers. (**J**) Organs weight in control and KO (≤ 10% body weight loss) normalized to whole body weight. (**K**) Daily food intake monitored in individualized cage (n = 19)/group. Data are presented as Mean ± SE, n = 19 (oil control), n = 20 (tamoxifen control), n = 19 (KO) mice/group. Data are presented as Mean ± SE (n = 4). *p < 0.05, **p < 0.001, ***p < 0.0001 from control (unpaired two-tailed Student’s t-test).

We employed a *Clec16a* tamoxifen inducible, ubiquitous knockout (KO; *Clec16a*^ΔUBC^) mouse model to study the role of *CLEC16A* in autoimmunity described previously^33^. Briefly, 10 weeks old mice were treated with tamoxifen to induce knockout. Recombination was confirmed at DNA and RNA level. Regular PCR of genomic DNA confirms removal of *CLEC16A* exon 3 in blood and various organs of *Clec16a*^*ΔUBC*^ mice (Fig. S1A). To assess extent of excision RT-PCR analysis from genomic DNA was performed. After recombination, *Clec16a*^*ΔUBC*^ mice retain 30% of exon 3 in comparison to control (Fig. S1B). Sanger sequence of cDNA from blood confirmed induced skipping of exon 3, frameshift of the reading frame and generation of STOP codons in exon 4 (Fig. S1C). The first visible observation we made in control and *Clec16a*^ΔUBC^ mice, fed a standard rodent diet, was a difference in body weight. *Clec16a*^ΔUBC^ mice exhibited severe weight loss, which deteriorated over the length of the study, starting 1 week after initiation of tamoxifen treatment, in comparison to tamoxifen treated control mice. Throughout the study, control mice showed a healthy appearance and maintained their body weight (Fig. 1A). To determine the cause of weight loss, *Clec16a*^ΔUBC^ and control mice were dissected and compared. Both male and female *Clec16a*^ΔUBC^ mice exhibited near to complete absence of typical gonadal white adipose tissue (gWAT) (Fig. 1B–D). Further examination indicated that all the WAT depots, including gonadal, inguinal, mesenteric, retroperitoneal, perineal, and pericardial, were remarkably reduced or absent in *Clec16a*^ΔUBC^ mice (Fig. 1B). We further confirmed the reduced expression of CLEC16A protein in gWAT, thymus, pancreas and splenocytes from *Clec16a*^ΔUBC^ mice by Western blot (Fig. 1E,F). The leftover CLEC16A depicted in KO is full-length protein that still retains the normal function. We hypothesized that reduced expression of *Clec16a* leads to dysregulation of ER homeostasis in adipose tissue in response to defective autophagy and elicits the lipolytic cascade.

To study if *Clec16a* KO evoked ER stress, we first examined gWAT for ER stress markers by RT-PCR (Table S1) and immunoblot analysis. As expected, the ER stress marker genes (GRP78, ATF6, IRE1a, XBP1 and CHOP) showed significant upregulation at mRNA levels in the KO mice (Fig. 1G). Immunoblot analysis further confirmed the above findings. gWAT lysates of KO mice showed significant upregulation GRP78, ATF6, XBP1 and CHOP with barely detectable phospho-IRE1a (Fig. 1H,I). We also checked the expression of the autophagosome marker LC3-I/II and P62 in gWAT lysate. *Clec16a*^ΔUBC^ mouse gWAT showed significant increase and accumulation of P62 and modest increase in LC3-II expression in comparison to tamoxifen treated control. These data confirmed ER stress was most likely activated in response to defective autophagic flux in adipose tissue of KO mice (Fig. 1H,I). To evaluate if weight loss in *Clec16a*^ΔUBC^ mice affected organ weight as opposed to only adipose loss, we measured thymus, heart spleen, pancreas, kidney, muscle and liver organ weight ratios in tamoxifen treated control and KO mice. Organ weight loss was not observed in KO mice with ≤ 10% body weight loss (Fig. 1J). Food intake was assessed next to evaluate whether this could explain the severe weight loss in *Clec16a*^ΔUBC^ mice. *Clec16a*^ΔUBC^ mice showed no significant increase or decrease in food consumption in comparison to the tamoxifen treated control mice, suggesting food consumption was not the reason behind the weight loss of KO mice (Fig. 1K). The body weight loss is strictly attributed only to the loss of adipose tissue. Thus, *Clec16a* loss in adipose promotes ER stress in response to defective autophagic flux. Persistent ER stress possibly activates the lipolytic cascade downstream resulting in body weight reduction leading to severe generalized lipodystrophy and a profound effect on all WAT deposits.

### Fat Lipolysis and severe metabolic abnormalities in *Clec16a*^ΔUBC^ mice

**Figure 2:**
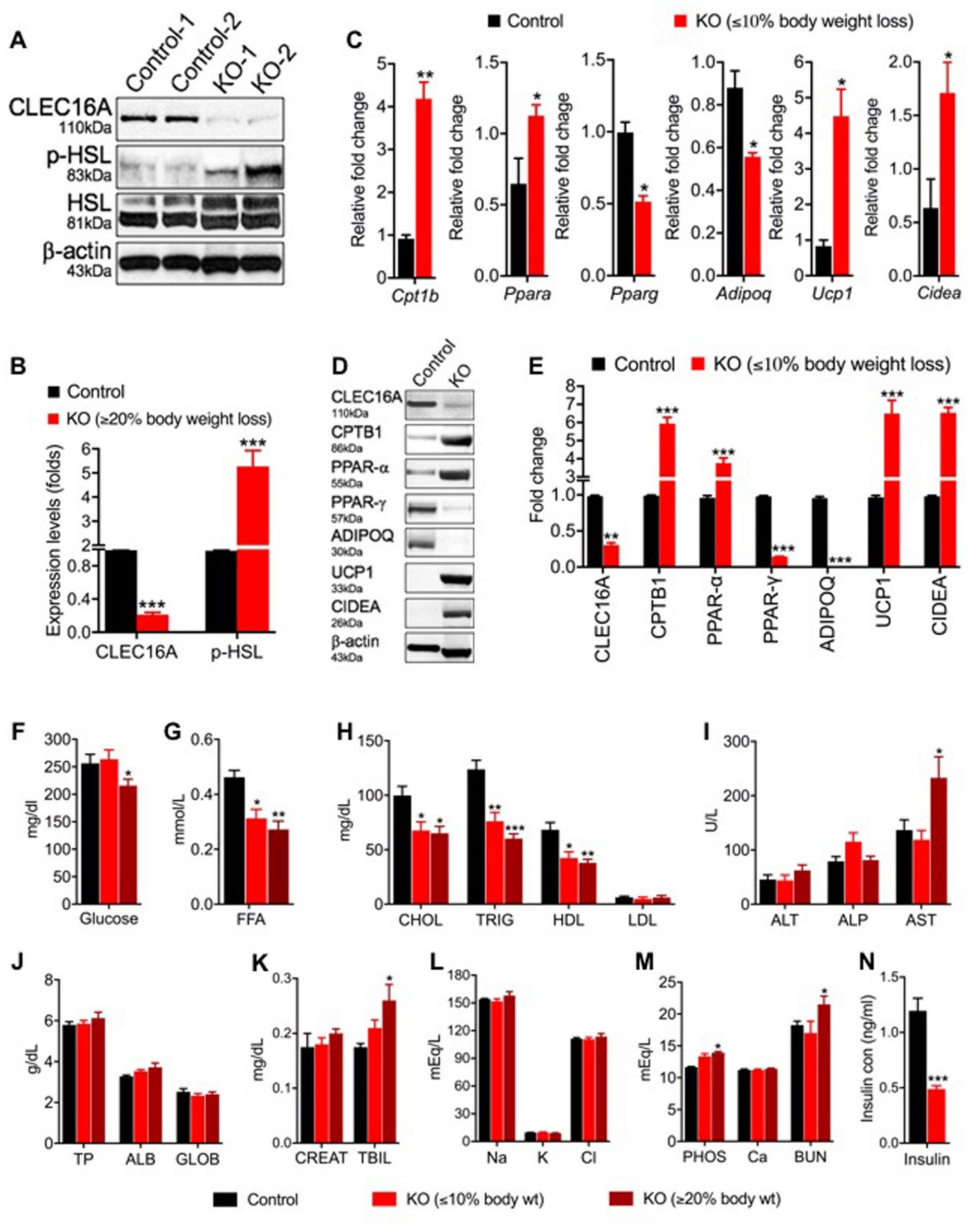
Fat lipolysis and severe metabolic abnormalities in *Clec16a*^*ΔUBC*^* mice*. (**A**) Representative immunoblot of HSL phosphorylation (Ser660) in control and *Clec16a*^*ΔUBC*^ (KO) gWAT (n = 3). (**B**) Graph depicts CLEC16A and p-HSL expression. Data presented as Mean ± SE of three independent repeats (**C**) The mRNA expression of lipid catabolism genes (*Cpt1b*, *Pparα*), adipogenic genes (*Pparγ* and *Adipoq*) and thermogenic genes (*Ucp1* and *Cidea*) from gWAT of tamoxifen control and *Clec16a*^*ΔUBC*^ mice determined by qPCR (n = 8 mice/group). Representative immunoblot (**D**) and graph (**E**) depicts CLEC16A, CPTB1, PPARα, PPARγ, ADIPOQ, UCP1 and CIDEA expression from gWAT of tamoxifen control and *Clec16a*^*ΔUBC*^ mice ≤ 10% body weight loss). Membranes were striped and reprobed for β-actin as a loading control. Data presented as Mean ± SE of three independent repeats (**F**) Serum blood glucose levels in 10-weeks-old tamoxifen control and *Clec16a*^*ΔUBC*^ (≤ 10% and ≥ 20% body weight loss) mice (n = 20/group). (**G**) Serum free fatty acid (FFA) levels (n = 20/group). (**H**) Serum cholesterol (CHOL), triglyceride (TRIG), high-density lipoprotein (HDL) and low-density lipoprotein (LDL) levels. (**I**) Serum alanine transaminase (ALT), alkaline phosphatase (ALP), aspartine aminotransferase (AST). (**J**) Serum total protein, albumin and globulin. (**K**) Serum creatinine and total bilirubin (TBIL) levels. (**L**) Serum electrolytes (Na, K and Cl). (**M**) Serum phosphorus (PHOS), calcium and blood urea nitrogen (BUN) levels. (**N**) Serum insulin in control and *Clec16a*^*ΔUBC*^ (≥ 20% body weight loss) mice. (**L**) Serum total bilirubin (TBIL) levels. Metabolite panel data are presented as Mean ± SE, n = 15 (tamoxifen control), n = 15 (KO) mice/group. Data presented as Mean ± SE of three independent repeats. *p < 0.05, **p < 0.001, ***p < 0.0001 from tamoxifen control (unpaired two-tailed Student’s t-test).

Next, we explored the mechanism behind the weight loss. Immunoblot analysis of *Clec16a*^ΔUBC^ mouse gWAT revealed a significant increase in phosphorylation of hormone sensitive lipase (HSL) as compared to tamoxifen treated controls (Fig. 2A,B).

To gain further insight in the mechanism(s) whereby *Clec16a* KO mediates its effect on energy expenditure, we measured expression of key genes regulating lipid metabolism in gWAT of *Clec16a*^ΔUBC^ mice (≤ 10% body weight loss) by RT-PCR (Table S1). Virtually no fat was left in mice exhibiting ≥ 20% body weight loss to analyze (Fig. 1B). We found carnitine palmitoyltransferase 1b (Cpt1b), a gene essential for adipose tissue fatty acid oxidation, was significantly upregulated in *Clec16a*^ΔUBC^ mouse gWAT, along with the upstream transcription factor, peroxisome proliferator-activated receptor alpha (Pparα). Further, the expression of the adipogenic gene, Pparγ and its downstream target, adiponectin precursor (Adipoq), were significantly reduced in *Clec16a*^ΔUBC^ KO gWAT. Thermogenic genes, including thermogenin (Ucp1), and the cell death-inducing DFFA-like effector A (Cidea) gene were significantly upregulated in WAT of *Clec16a*^ΔUBC^ KO mice (Fig. 2C). Importantly, the protein expression correlated with upregulated mRNA expression in gWAT of *Clec16a*^ΔUBC^ mice (Fig. 2D). Immunoblot analysis showed significant upregulation in expression of CPTB1, PPAR-α, UCP-1, CIDEA and significant downregulation in expression of PPAR-γ and ADIPOQ (Fig. 2D,E).

We also ran a detailed metabolite panel analysis on serum of control and *Clec16a*^ΔUBC^ KO mice to evaluate the possible metabolic shift leading to the lipodystrophic phenotype. For comparison we split KO mice in two groups, *Clec16a*^ΔUBC^ mice with ≤ 10% body weight loss and *Clec16a*^ΔUBC^ mice with ≥ 20% body weight loss, as virtually no fat was left in mice in the latter group. *Clec16a*^ΔUBC^ mice with up to 20% weight loss did not show any evidence of hyperglycemia (Fig. 2F). Lipid profiling in serum uncovered significant decreases in circulating free fatty acid (FFA) (Fig. 2G), cholesterol (CHOL), triglycerides (TG) and high-density lipoprotein (HDL) (Fig. 2H) in the *Clec16a*^ΔUBC^ mice in comparison to tamoxifen treated control mice. The reduction was more pronounced the greater the weight loss (Fig. 2H). Low-density lipoprotein (LDL) was unaltered (Fig. 2H). The livers of the *Clec16a*^ΔUBC^ showed no striking difference in gross morphology in comparison to control. Liver function tests, such as alanine transaminase (ALT), alkaline phosphatase (ALP), aspartine aminotransferase (AST) (Fig. 2I), total protein, albumin and globulin were unaltered (Fig. 2J). Total bilirubin (TBIL) was significantly increased in mice with ≥ 20% weight loss (Fig. 2K). Serum creatinine (Fig. 2K) and electrolytes showed no difference (Fig. 2L). However, phosphorus and blood urea nitrogen (BUN) were significantly upregulated in the KO mice depicting ≥ 20% body weight loss (Fig. 2M). Calcium (Ca) showed no change (Fig. 2M). *Clec16a*^ΔUBC^ mice with up to 20% weight loss showed significant reduction in serum insulin (Fig. 2N). Taken together, these data suggest that an activated lipolytic cascade in *Clec16a*^ΔUBC^ mice triggers fat loss and does not lead to hyperlipidemia and ectopic lipid accumulation. Thus, the severe weight loss in *Clec16a*^ΔUBC^ mice is associated with reduction in fat mass as a consequence of activated lipolysis and depicts a novel complex metabolic syndrome. Under chronically prolonged ER stress, compromised mitophagy and autophagy settings, *Clec16a*^ΔUBC^ mice undergo chronic metabolic remodeling through upregulation of catabolic and thermogenic genes, downregulation of downstream adipogenic genes and promote HSL-mediated lipolysis.

### *Clec16a*^ΔUBC^ mice display dysregulated JAK/STAT signaling and a robust inflammatory autoimmune phenotype

**Figure 3:**
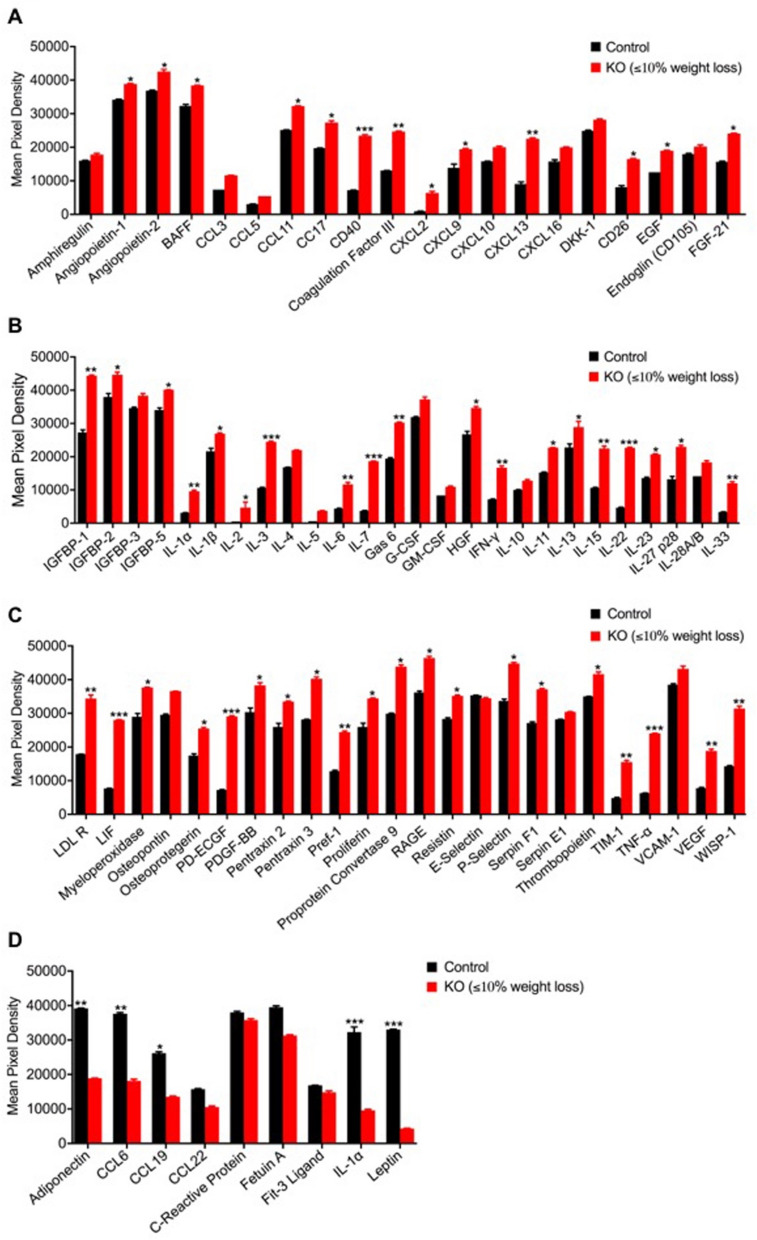

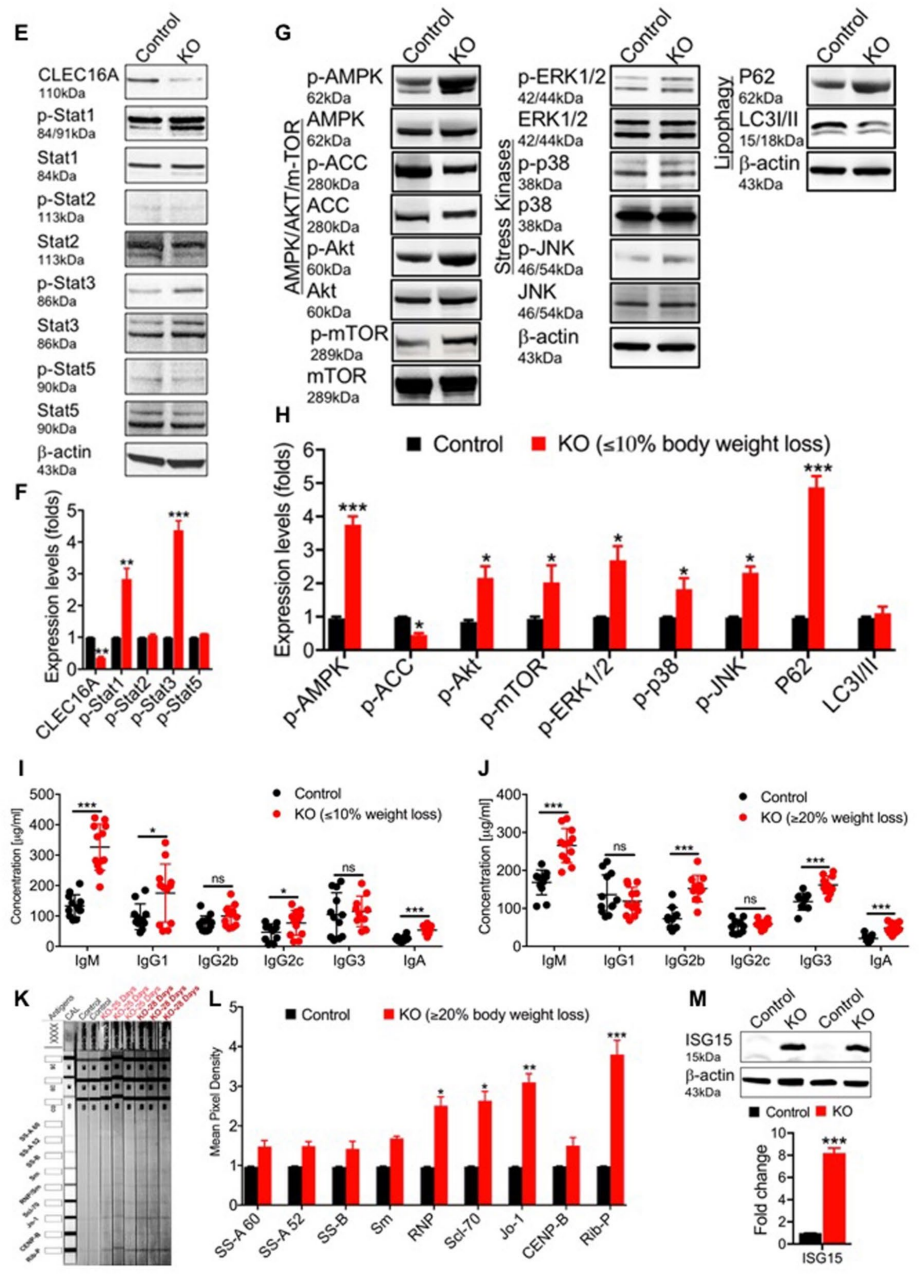
*Clec16a*^*ΔUBC*^* mice* gWAT exhibits dysregulated JAK/STAT signaling and inflammatory autoimmune phenotype. (**A**–**D**) The levels of plasma cytokines, adipokines, growth factors and other immune related proteins from gWAT lysate of control and *Clec16a*^*ΔUBC*^ (KO) mice (≤ 10% body weight loss). (**E**) Representative immunoblot depicts expression of CLEC16A, p-Stat1, Stat1, p-Stat2, Stat2, p-STAT3, Stat3, p-STAT5 and Stat5 in tamoxifen treated control and *Clec16a*^*ΔUBC*^ gWAT. (**F**) The graph depicts quantification of CLEC16A, and expression levels of phosphorylated p-Stat1, p-Stat2, p-Stat3, p-Stat5 relative to its basal level. (**G**) Representative immunoblot from gWAT of control, *Clec16a*^*ΔUBC*^ depicting expression levels of p-AMPK, AMPK, p-ACC, ACC, p-Akt, Akt, p-mTOR, p-ERK1/2, p-38, p38, p-JNK, JNK, P62 and LC3-I/II. Membranes were striped and reprobed for mTOR and actin as a loading control. (**H**) The graph depicts quantification and expression of phosphorylated p-AMPK, p-ACC, p-Akt, p-mTOR, p-ERK1/2, p-38, p-JNK relative to its basal levels, P62 and LC3-I/II. proteins. (**I**) Serum immunoglobulin and IgG subclass isotyping in sera of control and *Clec16a*^*ΔUBC*^ mice (≤ 10% body weight loss) (n = 11 mice/group). (**J**) Serum immunoglobulin and IgG subclass Isotyping in sera of control and KO mice with ≥ 20% body weight loss (n = 11mice/group). (**K**) *Clec16a*^*ΔUBC*^ induced autoantibodies. Serum samples from control and *Clec16a*^*ΔUBC*^ mice were assayed for antibodies to various nuclear antigens using a line assay Western blot. Lane 1 is the positive control showing all of the antigens; Lanes 2–3 are probed with sera from control mice; Lane 4–9 are from *Clec16a*^*ΔUBC*^ mice. (**L**) Quantitation graph depicts fold change for auto antibodies in control vs. *Clec16a*^*ΔUBC*^ mice sera. (**M**) Representative western blot and quantitation graph depicting significant upregulation of ISG15 in gonadal white adipose tissue of control and *Clec16a*^*ΔUBC*^ mice. Membrane was striped and reprobed for actin as a loading control. All data presented as Mean ± SE of three independent repeats. *p < 0.05; **p < 0.01; ***p < 0.001(unpaired two-tailed Student’s t-test). All data presented as Mean ± SE of three independent repeats. *p < 0.05; **p < 0.01; ***p < 0.001(unpaired two-tailed Student’s t-test).

Recently, we showed that incomplete mitophagy predisposes *Clec16a*^ΔUBC^ mice to a cascade of altered signaling functions resulting in pathogenic inflammation^33, 34^. In light of the above findings, we next evaluated the gonadal white adipose tissue (gWAT) from *Clec16a*^ΔUBC^ and control mice for cytokines, adipokines, growth factors and other immune related proteins to further delineate the impact of *Clec16a* loss (Figs. S2, S3 and Table S2). We observed significant upregulation of multiple cytokines, chemokines and growth factor genes, including several lipolytic cytokines (IL-1α, IL-1β, IL-2, IL-3, IL-6, IL-15, IFN-γ, and TNF-α (Fig. 3A–D), suggesting activation of an inflammatory lipolytic pathway in addition to the classical lipolytic pathway As anticipated, the loss of adipose tissue in the KO mice was further reflected by significantly decreased levels of adiponectin and leptin, two important adipokines that play a key role in lipid homeostasis and metabolism (Fig. 3D).

To determine the cause-and-effect of ER stress, lipolysis, and inflammation in adipose tissue of *Clec16a*^ΔUBC^ mice, we also performed immunoblot analysis in gWAT from *Clec16a*^ΔUBC^ mice for STATs phosphorylation in JAK/STAT signaling. gWAT from KO mice showed significant reduction in *CLEC16A* expression. Further, immunoblot analysis revealed significant increase in phosphorylation of p-STAT1 and p-STAT3 compared to control (Fig. 3E,F). No significant change was detected in p-STAT2 and p-STAT5 levels. AMP-activated protein kinase (AMPK) is an important sensor of nutrient levels and its activation in cells is associated with stimulation of fatty acid oxidation and glucose uptake. Our immunoblot analysis shows significant increase in phosphorylated AMPK and decreased phosphorylation of its target, acetyl-CoA carboxylase (ACC). Downstream, we observed significant increases in phosphorylated (p)-Akt, p-mTOR, p-38 and p-JNK oxidative stress-related kinases (Fig. 3G,H). We also probed adipose for two autophagy markers, P62/SQSTM and LC3-I/II. P62 is degraded by autophagy and accumulates upon inhibition of autophagy. LC3-II is a component of autophagosomes and is converted from LC3-I during autophagy. gWAT of KO mice exhibit significant accumulation of P62 (Fig. 3G,H). Our results suggest disrupted autophagy in adipose tissue triggers excessive inflammation via dysregulated ERK1/2, JNK, p38 and JAK/STAT signaling.

Next, we determined whether loss of *Clec16a* results in changes in serum immunoglobulin (Ig) isotypes, corresponding IgG subclasses were measured in both *Clec16a*^ΔUBC^ KO mice and tamoxifen treated control mice sera. We compared KO mice with ≤ 10% and ≥ 20% body weight loss to controls (Fig. 3I,J). *Clec16a*^ΔUBC^ KO mice with ≤ 10% body weight loss depicted significant changes in IgM, IgA and IgG subclass IgG1 and IgG2c levels, whereas *Clec16a*^ΔUBC^ mice with ≥ 20% body weight loss depicted significant upregulation in IgM, IgA, IgG2b and IgG3 IgG subclasses. IgG1 and IgG2c showed significant increase in mice at early stages of weight loss and were not significant in *Clec16a*^*ΔUBC*^ mice ≥ 20% body weight loss. IgM and IgA showed significant upregulation for both weight loss categories (Fig. 3I,J).

We further evaluated serum samples of *Clec16a*^ΔUBC^ (≥ 20% body weight loss) in comparison with control mice for antibodies to various nuclear antigens (ANAs) using ANA-9-Line Immunoblot assay (Fig. 3K,L). ANA-9-Line Immunoblot assay is a membrane-based enzyme immunoassay for the semi-quantitative measurement of IgG class autoantibodies to extractable nuclear antigens SS-A 52, SS-A 60, SS-B, RNP/Sm, Sm, centromere B, Jo-1, Scl-70 and ribosomal P proteins in serum or plasma. *Clec16a*^ΔUBC^ resulted in significant positive antibody titers for RNP/Sm, Scl-70, Jo-1 and ribosomal-P ANAs, indicative of systemic autoimmune disease in our *Clec16a*^ΔUBC^ mice. Taken together, our finding of positive antibodies and elevated serum Ig are indicative of excessive inflammatory responses and autoimmune progression in *Clec16a*^ΔUBC^ mice. We also evaluated interferon stimulated gene 15 (ISG15) expression in gWAT lysates, a well-known downstream target of STAT1. Under conditions of stress, ISGlyation acts as defense mechanism and signals a state of alert to induce a response by the immune system. *Clec16a*^ΔUBC^ mice gWAT lysates show significant upregulation of ISG15 (Fig. 3M). These serum Ig isotyping, cytokine, chemokine, and ISG15 results are indicative of excessive inflammatory responses in *Clec16a*^*ΔUBC*^ mice and indicate a role for *CLEC16A* in autoimmunity. These observations suggest a potential link between disrupted autophagy, ER stress and the presence of elevated inflammatory mediators in *Clec16a*^ΔUBC^ mice contributing to adipose atrophy and inflammation, affecting energy homeostasis and metabolism, which may contribute broadly to autoimmune diseases.

### The pan JAK inhibitor Tofacitinib and Rapamycin (mTOR inhibitor) partially rescue the lipodystrophic phenotype and improves survival of *Clec16a*^ΔUBC^ KO mice

**Figure 4:**
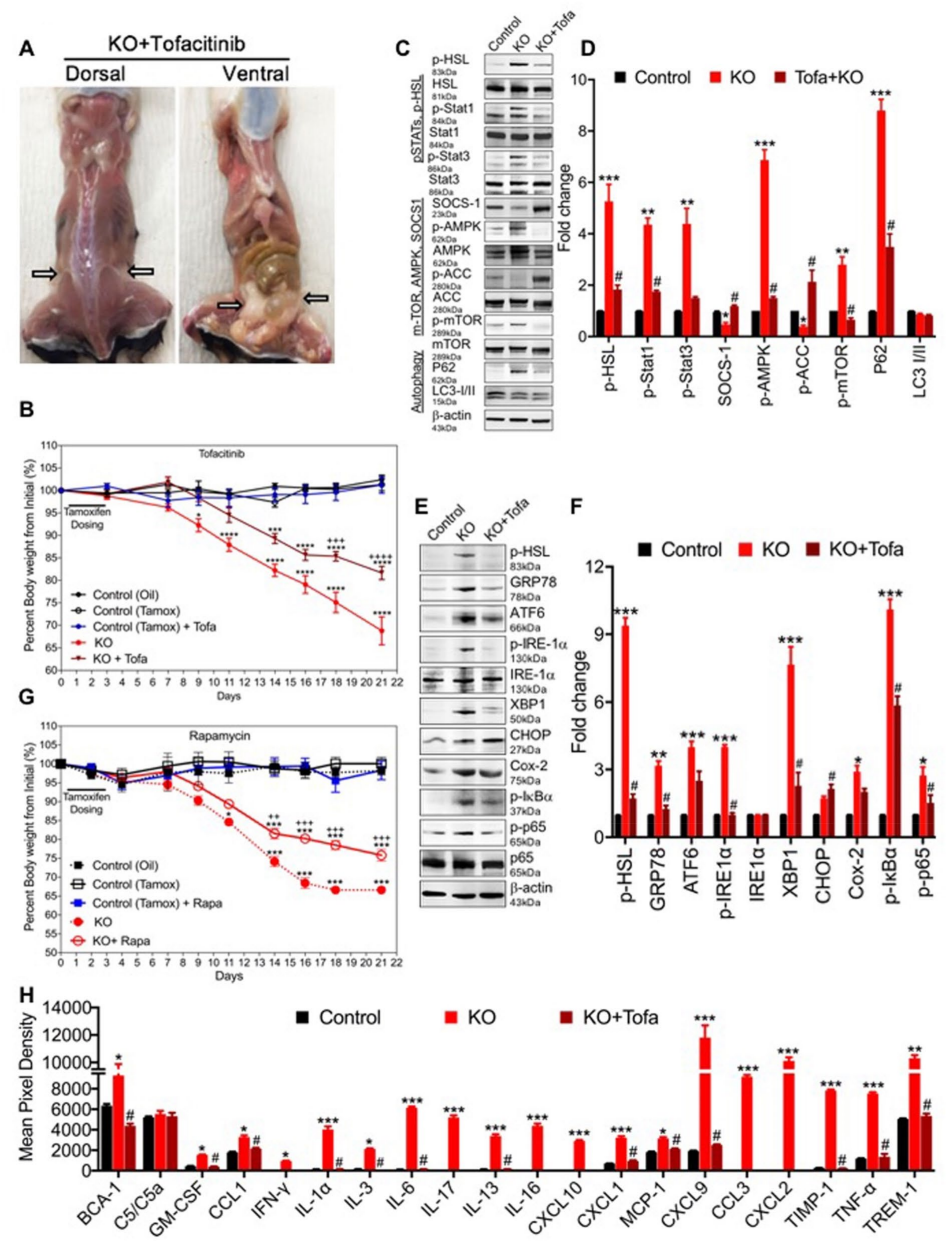
Tofacitinib and Rapamycin partially rescues the lipodystrophic phenotype and improve the survival of *Clec16a*^*ΔUBC*^ KO mice. (**A**) Representative dorsal and ventral dissection image depicting gross morphology and distribution of fat in Tofacitinib treated KO mice. (**B**) Body weight measured three times/week of tamoxifen control ± Tofacitnib and *Clec16a* KO ± Tofacitnib mice fed with standard chow. Data are presented as Mean ± SE, n = 15 mice/group. Two-way ANOVA. Tukey's multiple Comparison test, *p < 0.05, ****p < 0.0001 compared to the Control-tamoxifen vehicle group. ^+++^p < 0.001 and ^++++^p < 0.0001 compared to the KO group. (**C**) Representative western blot images from gWAT lysate of control (tamoxifen), KO, KO + Tofacitinib depicting expression levels of p-HSL (ser660), HSL, p-Stat, Stat1, p-Stat3, Stat3, p-AMPK, AMPK, p-ACC, ACC, p-mTOR, P62 and LC3-I/II. Membrane was striped and reprobed for mTOR, HSL and β-actin as a loading control. (**D**) Quantitation graph depicts fold change expression levels of p-HSL, p-STAT1, p-STAT3, p-AMPK, p-ACC, p-mTOR, P62 and LC3-I/II from control (tamoxifen), KO, KO + Tofacitinib. (**E**) Representative immunoblot depicting protein expression of p-HSL (Lipolysis), ER stress markers (GRP78, ATF6, p-IRE-1α, IRE-1α, XBP1, CHOP), COX-2 and p-IκBα, p-p65 and p65 in control, KO + Tofacitinib gWAT lysates. Membrane was stripped and probed for β-actin as loading control. (**F**) Graph depicts tofacitinib mediated rescue in gWAT. (**G**) Body weight measured three times/week of control ± Rapamycin and *Clec16a* ± Rapamycin mice fed with standard chow. Data are presented as mean ± SE, n = 15 mice/group. (**H**) Predominant Th-1 cytokine/chemokine in *Clec16a*^*ΔUBC*^ KO mice and rescue with Tofacitinib. The representative graph is the quantification of cytokines and chemokine from plasma of tamoxifen control ± Tofacitnib and *Clec16a* ± Tofacitnib mice using the Mouse Cytokine Array panel. The amount of BCA-1, C5/C5a, GM-CSF, CCL1, IFN-γ, IL-1α, IL-3, IL-6, IL-17, IL-13, IL-16, CXCL10, CXCL1, MCP-1, MIG (CXCL9), CCL3, CXCL2, TIMP-1, TNF-α and TREM-1 in the plasma are reported. The results are presented as average signal (pixel density) of the pair of duplicate spots representing each cytokine or chemokine analyzed using Image-J software. Data expressed as Mean ± SE of three independent experiments. *p < 0.05; **p < 0.01; ***p < 0.001 from control (unpaired two-tailed Student’s t-test). ^#^p < 0.001 Tofacitinib vs. KO (unpaired two-tailed Student’s t-test).

Recent discoveries support the emerging view that autoinflammatory diseases may be due to pathological derangement of stress sensing pathways that normally function in host defense. The 16p13 chromosomal region where CLEC16A is situated has been focus of several autoimmune diseases. Two immune-regulatory genes of potential interest for autoimmunity i.e., the major histocompatibility complex (MHC) class II transactivator (*CIITA*) gene and the suppressor of cytokine signaling 1 (*SOCS1*) gene, are located close to *CLEC16A* (Fig. S4A,B). *CLEC16’s* genomic location next to the *SOCS1* gene (important for immune cell homeostasis and regulation of inflammation by modulating the JAK/STAT pathway), and *CLEC16’s* expression specificity in immune cells, makes it an ideal candidate to be explored as potential druggable target. We treated *Clec16a*^ΔUBC^ mice with the pan JAK inhibitor tofacitinib; tofacitinib treatment significantly attenuated the fat and weight loss and improved the survival of *Clec16a* KO mice (Fig. 4A,B). Tofacitinib-treated control mice remained healthy and maintained their body weight throughout the study.

To address the underlying mechanism behind the tofacitinib rescue effect, we performed RT-PCR for SOCS1 and SOCS3 expression and immunoblot analysis on gWAT isolated from controls, *Clec16a*^ΔUBC^ without treatment, and *Clec16a*^ΔUBC^ tofacitinib-treated mice. We evaluated p-HSL, p-STAT1, p-STAT3, SOCS-1, AMPK, mTOR, P62 and LC3I/II and expression (Fig. 4C,D). SOCS1 mRNA expression from gWAT of tofacitnib-treated *Clec16a*^ΔUBC^ mice showed significant upregulation in comparison with untreated *Clec16a*^ΔUBC^ mice (Fig. S5A). SOCS3 expression showed no significant difference between the groups (Fig. S5B). Immunoblot analysis revealed significant upregulation of phospho-HSL in *Clec16a*^ΔUBC^ and reduction in the tofacitinib-treated *Clec16a*^ΔUBC^ mice. Examination of adipose tissue in *Clec16a*^ΔUBC^ mice demonstrated upregulation of p-STAT1 and p-STAT3. Tofacitinib downregulated both p-STAT1 and p-STAT3 to control levels. We also observed a significant increase in phosphorylation of AMPK. However, its target, ACC, exhibited reduced phosphorylation in the *Clec16a*^ΔUBC^ mice. Tofacitnib treatment significantly reduced the p-AMPK and promoted phosphorylation of ACC. Another downstream effector of AMPK is mTOR signaling that regulates a plethora of functions, including autophagy. Significant accumulation of P62 and a significant increase in phosphorylation of mTOR in gWAT of *Clec16a* mice was observed. Tofacitinib treatment decreased these levels. (Fig. 4C,D). We also evaluated lipolytic, ER stress, and inflammatory related proteins in tofacitinib-treated KO mice (Fig. 4E,F). Tofacitinib-treated KO mice exhibited significant downregulation in p-HSL, and ER stress proteins (GRP78, ATF6, p-IRE1a, XBP1 and CHOP). Tofacitnib treatment reduced expression of COX-2 and p-IκBα significantly as well. In light of elevated ER stress and dysregulated autophagy in adipose tissue of *Clec16a*^ΔUBC^ mice, we evaluated treatment with rapamycin (autophagy inducer). Rapamycin inhibits mTOR signaling stimulating mitophagy/autophagy through AMPK and ULK1 activation. Rapamycin treatment significantly reduced severe weight loss and improved the survival of *Clec16a*^ΔUBC^ mice similar to tofacitinib (Fig. 4G). During the same time period control ± Rapamycin mice showed a healthy appearance and maintained their body weight throughout the study in comparison to *Clec16a*^ΔUBC^ mice. Further, quantitation of cytokines and chemokines in plasma of *Clec16a*^ΔUBC^ KO showed robust upregulation of IFN-γ, IL-1α, IL-3, IL-6, IL-13, IL-16, TNF-α, several monocyte/macrophages chemoattractant proteins and IL-17 in comparison to control. We observed near complete reversal of the inflammatory cytokines and chemokines in the tofacitinib-treated mice compared to *Clec16a*^ΔUBC^ without treatment (Fig. 4H, Fig. S5C,D). Taken together, tofacitinib exerts its multifaceted effect on HSL-mediated lipolysis, AMPK, mTOR, JAK-STATs, and autophagy/lipophagy and ER stress signaling, improves survival, and rescues the inflammatory lipodystrophic phenotype exhibited by *Clec16a*^ΔUBC^ mice.

**Figure 5:**
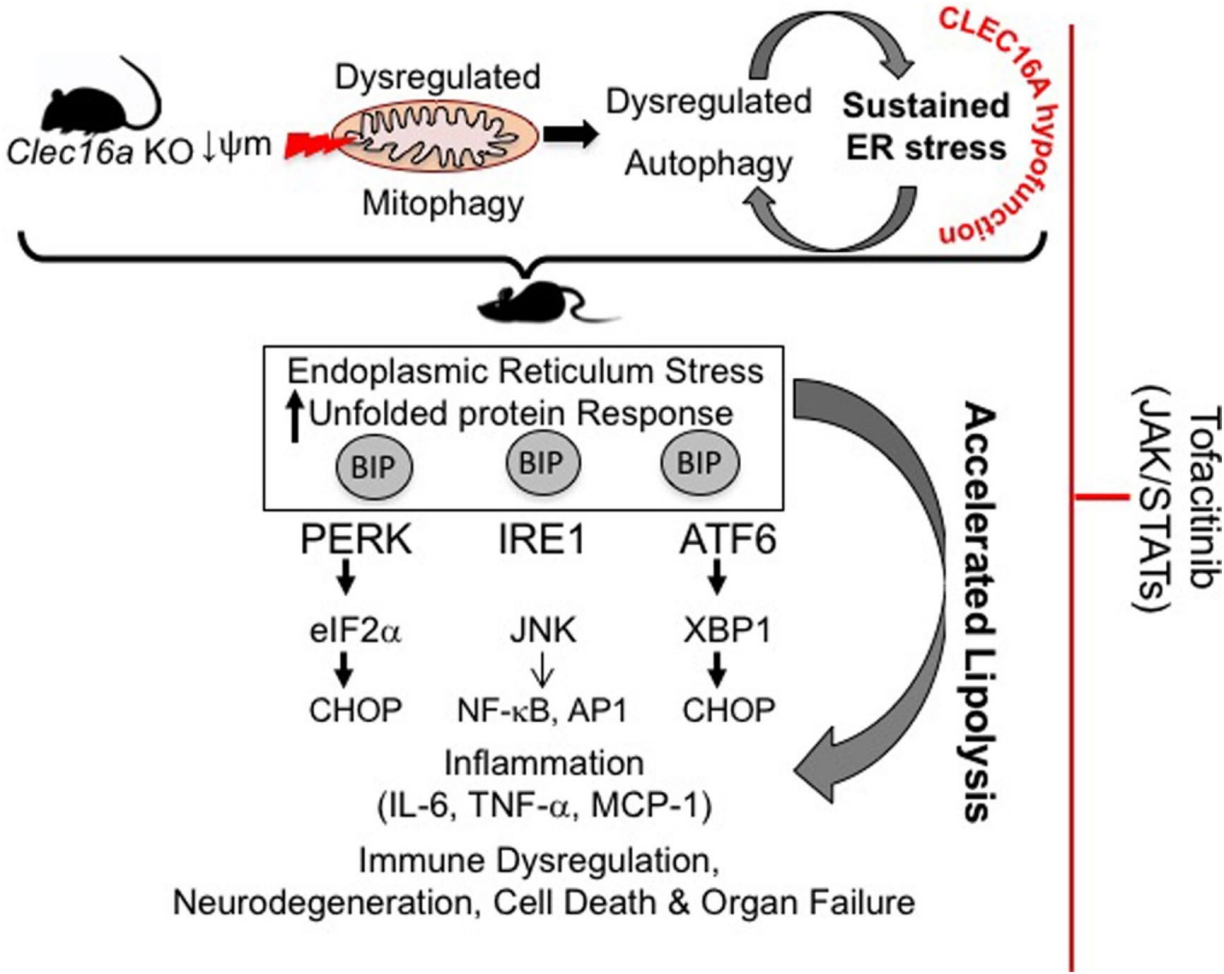
Tofacitnib mediated partial rescue of *Clec16a*^*ΔUBC*^ KO phenotype. Model depicting loss of *Clec16a* leads to a vicious cycle of autophagic impairment and ER stress. ER stress activates lipolytic cascade. Excessive HSL-driven lipolysis stimulates inflammation in adipose tissue of KO mice. Tofacitinib exerts its multifaceted effect on HSL-mediated lipolysis, AMPK, mTOR, JAK-STATs, and autophagy/lipophagy and ER stress signaling, improves survival, and rescues the inflammatory lipodystrophic phenotype exhibited by *Clec16a*^*ΔUBC*^ KO mice.

In conclusion, our whole-body inducible *Clec16a*^ΔUBC^ mouse model provides an excellent tool to address the mechanism by which the *CLEC16A* risk-associated variants may lead to autoimmune/inflammatory and lipodystrophic phenotypes (Fig. 5). Our results from the JAK pan-inhibitor-(tofacitinib) and the selective autophagy inducer, rapamycin, suggest that in patients harboring variants that result in *CLEC16A* hypofunction, drugs with modulatory effects on ER stress, mitophagy/autophagy/SOCS1-JAK-STAT signaling could compensate for the attenuated *CLEC16A* activity and present formidable candidates for targeted interventions resulting in a balanced energy homeostasis.

## Discussion

Our study of the whole-body, inducible *Clec16a*^ΔUBC^ mouse model demonstrates that loss of CLEC16A function leads to a vicious cycle of autophagic impairment, ER stress, and activation of lipolytic cascade in adipose tissue. This in turn contributes to the severe weight loss accompanied by a systemic inflammatory response, key metabolic perturbations, altered lipid metabolism and inflammatory lipodystrophic phenotype. Unlike other established models of lipodystrophy, our *Clec16a*^ΔUBC^ mice do not exhibit hypertriglyceridemia and hepatic steatosis, implying that we may have a novel phenotype different from known mammalian models of lipodystrophy^49^. As *CLEC16A* has been genetically linked with multiple autoimmune disorders, it presents an attractive candidate for studies addressing potential therapeutic approaches for inflammation and autoimmune disorders.

Previous studies have highlighted the role of *CLEC16A* as critical regulator of mitophagy^23^ and autophagy^48^. *CLEC16A* is a ubiquitously expressed gene with functions in pancreatic β-cells, immune cells and neurons. *CLEC16A* encodes an E3 ligase that promotes NRDP1 ubiquitination^23–25^. An effect of disease-related CLEC16A SNPs to influence CLEC16A expression or the expression of nearby related genes in immune cells remains controversial^22^. While *CLEC16A* and NRDP1 both play vital roles within immune cells^24, 55^, the function of CLEC16A SNPs (on mitophagy or other processes) within other human cell types requires future investigation.

Our work in mice illustrates that downregulation of CLEC16A either via genetic KO or via siRNA-mediated knockdown results in dysregulated mitophagy and cell death and predisposes *Clec16a*^ΔUBC^ KO mice to a cascade of altered signaling functions resulting in a pathogenic systemic inflammatory response^33^. Recent work from our laboratory demonstrates the presence of a derailed functional link between CLEC16A and NK cells. Indeed, *Clec16a*^ΔUBC^ mice show altered immune cell populations, increased splenic NK cell cytotoxicity, imbalance in dendritic cell subsets, altered receptor expression, upregulated cytokine and chemokine secretion^34^. Altered immune cell populations, increased killing, upregulated cytokines/chemokine secretion, together with an imbalance in dendritic cell subsets observed under previously established disrupted mitophagy conditions, perpetuates cell death and tissue destruction. This provokes inflammatory reactions potentially impacting progression and pathogenesis of various autoimmune and inflammatory diseases. The resulting cell death constitutes a source of novel antigens which can further stimulate the autoimmune response. This is further supported by the fact that conditional targeting of T cells in CD4 Cre *Clec16a*^loxP^ mice showed no difference in T cell repertoire, metabolism, lipolysis, inflammation or pathological phenotype^33^.

In the current study, evaluation of autophagy in *Clec16a*^ΔUBC^ WAT revealed accumulation of P62, indicative of inhibition of autophagy/lipophagy. Excessive adipocyte lipolysis possibly generates lipid mediators and triggers further inflammation. We show an upregulation of all three key sensors (PERK, ATF-6 and IRE1a) of the UPR pathway in *Clec16a*^ΔUBC^ mice with a picture of humanoid-like metabolic syndrome and autoimmunity, with potential implications for understanding mechanisms of weight reduction for future interventions. Dysregulated lipolytic activation in our model through JAK/STAT-SOCS, p44/42 MAPK and JNK pathways derails the normal physiologic response adding further insult under compromised mitophagy in *Clec16a*^ΔUBC^ mice. The near complete loss of adipose tissue in the KO mice was accompanied by decreased levels of adiponectin and leptin, two important adipokines that play a key role in lipid homeostasis and metabolism. The observed increase in liver function enzymes, suggestive of recent or ongoing liver cell damage, is in keeping with the notion that *Clec16a*^ΔUBC^ mice depict a complex metabolic syndrome and undergo a chronic metabolic remodeling response. *Cpt1b*, a gene essential for adipose tissue fatty acid oxidation, was significantly upregulated in *Clec16a*^ΔUBC^ mouse iWAT and expression of its upstream transcription factor, *Pparα*, is also upregulated. In contrast expression of the adipogenic gene, *Pparγ* and its downstream target *Adipoq*, were significantly reduced in KO gWAT. Moreover, there was significant upregulation of the thermogenic genes, *Ucp1* and *Cidea*, in gWAT implying that immune cells respond to this metabolic shift and sense them as stress. In general, weight loss attributed to caloric restriction is unrelated to and occurs in the absence of inflammation^54^. The presence of specific autoantibodies, elevated Ig isotyping levels together with induced upregulation of multiple cytokines, chemokines and growth factor genes, including several lipolytic cytokines suggest activation of inflammatory lipolytic pathway in addition to the classical lipolytic pathway, are indicative of autoinflammatory response in *Clec16a*^ΔUBC^ mice. Thus, in our *Clec16a*^ΔUBC^ mice mitophagy, lipophagy and JAK/STAT-SOCS signaling are affected presenting concomitant lipolysis and metabolic derailments and contributing to the pathogenesis of autoimmunity.

Tofacitinib, a pan JAK/STAT inhibitor with immunomodulatory and anti-inflammatory activities^56, 57^ binds to JAK and prevents the activation of the JAK/STAT signaling pathway. Our results show tofacitnib exerts its multifaceted effect on AMPK, mTOR, HSL, autophagy, JAK-STAT mediated SOCS signaling and partially reduces the lipodystrophic phenotype. Treatment suppresses the expression of ER stress response genes and inflammatory cytokines and improves survival of *Clec16a*^ΔUBC^ mice by modulating ER stress caused by excessive lipolysis to repurpose them for novel clinical indications in future human studies.

Partial improvement of the phenotype could be explained by the fact that receptors for cytokines like TNF-α are structurally distinct from Type I/II cytokine receptors and are independent of JAKs for signaling and persistent ER stress in response to compromised mitophagy and autophagy. An additional level of complexity in the JAK controlled network is that signals from most cytokines involve two different JAKs. Rapamycin treatment also partially reverses the phenotype and improves survival of *Clec16a*^ΔUBC^ mice similar to tofacitinib. Recent studies implicate a role for the cellular UPR pathways, nucleic acid signaling and ER stress in autoimmune and autoinflammatory disorders on multiple level in a wide variety of tissue that divide slowly, such as neurons, glial cells and pancreatic-β cells leading to inflammation and cell death^39, 51, 58, 59^. ER stress-driven inflammation can either promote or impede disease via inflammatory pathways depending on the cell type, disease stage, and type of ER stressor^59–61^. Also, topical or systemic therapeutic targeting of ER stress mediated inflammation might be beneficial for the target cells but detrimental for the immune cells, and vice versa. Therapeutic targeting of ER stress components in immune cells is particularly difficult because intact UPR signaling seems to be essential for their development and proper functioning^61^. ISG15 also plays an important role in autophagy, interacts with p62 and histone deacetylase 6 (HDA6), and promotes clearance of protein aggregates^62^. Upregulated ISG15 expression indicates that possibly CLEC16A directly/indirectly modulates IGS15 expression and immune response through regulation of the Type I IFN-STAT signaling under stress and orchestrates a state of alert. ISG15 appears to not inhibit but rather enhance the secretion of cytokines to counteract the threat. Our previous studies from *Clec16a*^ΔUBC^ mice^33, 34^ and results from the current study support the “spill over” hypothesis of primary disturbance of the innate immune system into autoimmunity. Further studies are needed to fully rescue the observed defect by use of one or more of the specific ER stresses, autophagy/mitophagy/Type I IFN-STAT signaling modulators alone or in combination.

Taken together, *Clec16a*^ΔUBC^ mice display extensive loss of body fat despite no decrease in food intake, together with compromised mitophagy and induction of systemic inflammatory response involving multiple cytokines/chemokines via inflammatory and classical lipolytic pathways. Our findings support the role of UPR and ER stress in sensing danger and its contribution to aberrant immune response in autoimmune and autoinflammatory diseases. Much of the research conducted to date to understand the mechanism of autoimmunity has been performed in animal models that are induced through transfer of adaptive immune populations, or by intentionally breaking tolerance, and are insulated from both the genetic and environmental influences Harmful imbalances in autophagic regulation are conceptualized as a state of autophagic stress in many human diseases and failure to remove dysfunctional mitochondria leads to hyper-activation of inflammatory pathways and enhancement of the inflammatory function in major auto-inflammatory and autoimmune diseases^63–66^. The *Clec16a*^ΔUBC^ mouse represents a unique model to study the role of *Clec16a* and associated autophagy/mitophagy defects leading to autoinflammatory, lipodystrophy and neurodegeneration (Hain et al., under review).

Given *CLEC16A* SNP’s association with several autoimmune disorders and its selective expression in immune cells (Fig. S4, Supplementary Table 3), patient populations harboring variants that result in CLEC16A hypofunction, our results suggest that drugs with modulatory effects on ER stress, lipophagy/autophagy/mitophagy, or inflammatory pathways could compensate for the attenuated CLEC16A activity and present formidable candidates for targeted interventions in autoimmunity. Indeed, treatment of *Clec16a*^ΔUBC^ mice with the pan-JAK/STAT inhibitor tofacitinib, partially rescues the pathological lipodystrophic phenotype and improves survival. Further exploitation of this unique *in-vivo* model may also unveil new directions in treatment and interventions of lipodystrophy, obesity, metabolic and age-associated inflammation research.

## Methods

### UBC-Cre-Clec16a^loxP^ (*Clec16a* KO) mice

The Institutional Animal Care and Use Committee (IACUC) of the Children’s Hospital of Philadelphia approved all animal studies. All methods were performed in accordance with the IACUC guidelines and regulations. All study was carried out in compliance with the ARRIVE guidelines. UBC-*Cre-Clec16a*^loxP/loxp^ mice were generated as described previously^33^. Genotyping primer sequences used were: Clec16a-flox-Forward 5′-TGTGTTGTTCTCCCTTGCAG-3′, Clec16a-flox-Reverse 5′-GAATAGTGGGCAAACACACGCCACTA-3′, UBC-Cre-Forward 5′-CGCTCGGGGTTGGCGAGTGTGTTTTGT-3′, UBC-Cre-Reserve 5′-GCCTGGCGATCCCTGAACATGTCCATC-3. Mice were group-housed on an individually-ventilated cage rack system on a 12:12 light:dark cycle. Mice were fed standard rodent chow and water ad libitum. Ten-week-old mice were treated with tamoxifen (100 mg/kg/day) to induce knockout of *Clec16a* (*Clec16a*^ΔUBC^ KO). Control groups of mice were treated with vehicle (10%: ethanol: 90% corn oil) or tamoxifen (100 mg/kg/day) by gavage at 24-h intervals for 4 consecutive days. Mice were sacrificed by CO_2_ asphyxiation. Wherever specified KO mice were compared to tamoxifen treated controls. Perigonadal adipose tissue, thymus, spleen and pancreas were excised and weighed. Tissue samples were flash frozen in liquid nitrogen and stored at − 80 °C. Excised spleens were washed in chilled PBS, teased and then compressed between frosted glass slides and washed with 1XPBS. Single cell suspension was prepared by passing the suspension through sterile mesh. The cells were then counted by hemocytometer. Basal and terminal glucose readings were determined by glucometer readings (AlphaTrak II, Zoetisus) from tail punctures. *Tofacitinib treatment* Tofacitinib was purchased from Med Chem Express (Cat. HY-40354/CS-0050). Tofacitinib was formulated in dimethyl sulfoxide (DMSO) and diluted in sterile saline (0.9% NaCl). Final concentration of DMSO in working solution was 0.74%. Mice received a 15 mg/kg dose of Tofacitinib bid via the intraperitoneal cavity (10 ml/kg) for 21 days. *Rapamycin treatment *Rapamycin was purchased from Alfa Aesar (Cat. J62473) and was formulated in DMSO, diluted with 5% PEG 300 and 5% Tween 80 in saline. Final concentration of DMSO in working solution was 0.4%. A 1 mg/kg dose of rapamycin was initially administered intraperitoneally (10 ml/kg ip) to the mice for 5 straight days and then every other day after the fifth dose.

### Food intake study

Mice were housed two animals per cage for 1 week prior to the start of the experiment. Standard pelleted rodent chow from LabDiet 5015 (Purina Mills, Richmond, IN, USA) was used for the food intake study. Food was weighed and placed into the food hoppers of each cage. Ad libitum requirements were met by placing sufficient quantity of food into the hopper so that the hopper did not run empty between measurements. Every 2–3 days, food was removed and weighed along with food spillage which was meticulously collected from each cage. After every measurement, mice were placed into a fresh clean cage. Food intake was calculated by ((FOOD IN(g) − (FOOD OUT(g) + SPILLAGE(g)))/number of mice per cage)/number of days.

### Serum metabolite analysis

Briefly, blood collected into MiniCollect Serum tubes from fed *Clec16a* KO with ≤ 10% and ≥ 20% body weight loss (n = 10) and corresponding tamoxifen treated control groups (n = 12) was allowed to clot for 30 min. Samples were spun by centrifugation at 3000*g* for 10 min at 4 °C to collect the serum fraction. Collected serums were snap frozen in liquid nitrogen, stored immediately at − 80 °C and shipped on dry ice to Charles River Laboratories for analysis. The serum samples were analyzed for circulating free fatty acid (Non-esterified Fatty Acid, NEFA), cholesterol (CHOL), high-density lipoprotein (HDL), low-density lipoprotein (LDL), triglycerides (TRIG), total protein (TP), alanine transaminase (ALT), albumin (ALB), alkaline phosphatase (ALP), AST (aspartine aminotransferase), total bilirubin (TBIL), glucose (GLU), blood urea nitrogen (BUN), creatinine CREAT, Calcium (Ca), potassium (K), phosphorus (PHOS), sodium (Na) (Olympus AU640e analyzer, Olympus), and Cl (Beckman Coulter) & FFA (Wako). The data was generated directly from the analyzer, and then formatted in an Excel template for reporting purposes.

### Insulin ELISA

Crystal Chem’s Ultra-Sensitive Mouse Insulin ELISA Kit was used to evaluate serum Insulin as per manufactures instructions. Briefly, 5 μL serum sample was diluted with 95 μL diluent and added to the precoated wells. Plate was incubated for 2 h at 4 °C. After wash, 100 μL conjugate solution was added and incubated for another 30 min at room temperature. After three washes 100 μL of substrate solution was added. Following another incubation for 40 min, 100 μL stop solution was added. Optical density was measured at 450/630 nm using a Synergy HT Microplate Reader (BioTek).

### RNA extraction quantitative real-time PCR

Total RNA was isolated with Trizol reagent (Invitrogen) following RNA purification using the RNeasy Mini Kit (Qiagen) and converted to cDNA by the High Capacity RNA-to-cDNA Kit (Applied Biosystems) according to the manufacturer’s protocols as described previously^34^. Fluorescence-based real time PCR was performed using the Fast SYBR Green Mater Mix (Applied Biosystems) in triplicates for each sample. All assays had primers covering exon-exon boarders to avoid DNA contamination. The PCR runs were performed on ViiA 7 Real Time PCR System using ViiA7 RUO software v1.2.2 (Life Technologies).

### Western Blot

Briefly, lysis was performed with NP40 lysis buffer. Harvested adipose tissues were lysed with a glass Dounce homogenizer in NP40 lysis buffer [50 mM Tris–HCl pH (pH 7.4), 150 mM NaCl, 1% NP-40, 1 mM EDTA, 10 mM NaF, 10% glycerol, and protease inhibitors] and then centrifuged at 13,000 rpm, 4 °C for 10 min to obtain a postnuclear supernatant fraction. Protein concentrations were determined by the Bradford method for normalization of protein loading. The lysates were electrophoresed on 4–12% NuPAGE Bis–Tris gels in MOPS SDS running buffer and transferred onto nitrocellulose membranes (Invitrogen) overnight. The membranes were blocked in 3% BSA and incubated with indicated primary antibodies where specified for: CLEC16A, p-HSL (ser660), HSL, p-Stat1, Stat1, p-Stat2, Stat2, p-Stat3, Stat3, p-Stat5, Stat5, p-AMPK, AMPK, p-ACC, ACC, p-Akt, Akt, p-mTOR, mTOR, p-ERK1/2, ERK1/2, p-p38, p38, p-JNK, JNK, P62, LC3-I/II and ISG15 (Cell Signaling Technology), SOCS-1(Abcam) and β-actin (Santa Cruz). ER stress was evaluated using ER Stress/UPR Antibody Pack (Novus Biologicals): GRP78/HSPA5 (NBP1-06277SS), IRE1 alpha (p Ser724) (NB100-2323SS), IRE1 alpha (NB100-2324SS), XBP1 (NBP1-77681SS), ATF6 (70B1413.1) (NBP1-40256SS), CHOP/GADD153 antibody (NBP2-13172SS)^67^. Lipolysis activation Antibody Sampler Kit was used to evaluate p-HSL (ser660) and total HSL^68^. Phospho-Stat Antibody Sampler kit (Cell Signaling Technology), was used to evaluate p-STAT’s^69^. p-mTOR and mTOR was evaluated using mTOR Substrates Antibody Sampler Kit (Cell Signaling Technology)^69^. P-AMPK and p-ACC was evaluated using AMPK and ACC Antibody Sampler Kit (Cell Signaling Technology)^70^. The membranes were washed and incubated with a respective secondary antibody and bound antibody was detected with WesternBright ECL kit (Advansta). Membranes were stripped and re-probed for actin as loading control. Band intensities were measured using Image J software (NIH Shareware), scanned in grey scale mode at 300 DPI and saved in TIFF format, measuring the area under each peak for each band.

### Serum immunoglobulin enzyme-linked immunosorbent assay (ELISA)

Briefly, serum was isolated from control (tamoxifen) and KO (≤ 10% body weight loss) mice. The concentrations of Ig subclasses in mouse sera were determined using isotype specific antibodies with a sandwich ELISA protocol according to the manufacturer’s protocols as described previously^71^. Monoclonal anti-mouse IgA-HRP, IgM-HRP, IgG1-HRP, IgG2b-HRP, IgG2c-HRP and IgG3-HRP were purchased from SouthernBiotech. These were used in two-fold serial dilutions starting at 10 ng/ml. Flat-bottom, 96-well plate (Nunc) was coated with capture antibody overnight at 1:250 dilution. Plates were blocked with 10% FCS in PBS buffer for 2 h and incubated with sample serum (1:10,000 for IgA and IgM and 1:20,000 for IgG1, IgG2b, IgG2c and IgG3) for 2 h at room temperature and detected with HRP-conjugated Ig subclass antibody (SouthernBiotech, 1:10,000 dilution) for 1 h at room temperature. Plates were developed with TMB substrate solution (eBioscience) and read at 450 nM using a Synergy HT Microplate Reader (BioTek).

### ANA line blot assay

Specific autoimmune targets were identified by immunoblotting on nitrocellulose bound with known nuclear antigens (ANA-9-Line, ORGENTEC Diagnostika GmbHCarl-Zeiss, Mainz-Germany), according to manufacturer instructions, modified to detect mouse IgG using a goat anti-mouse IgG secondary antibody conjugated to alkaline phosphatase as described previously^72, 73^. Briefly, the serum samples were diluted 100-fold and incubated on blocked strips for 60 min, then washed and developed with the alkaline phosphatase (AP)-conjugated goat anti-mouse IgG secondary antibody. The bands were visualized using the AP substrate solution provided with the kit. Positive reactivity was determined by comparing reactive bands with a positive control.

### Mouse cytokine array

The Proteome Profiler Mouse XL Cytokine Array Kit, (ARY028, R&D Systems) was used to quantify the 111 mouse proteins (cytokines, chemokines and growth factors) from gWAT of control (tamoxifen) and KO (≤ 10% body weight loss) mice according to the manufacturer's instructions as described previously^74^. Proteome Profiler Mouse Cytokine Array Kit, Panel A (ARY006, R&D Systems) was used to quantify 33 chemokine and cytokines in plasma of control and KO ± Tofacitinib according to the manufacturer's instructions as described previously^75, 76^. Briefly, gonadal adipose lysate/plasma was diluted and mixed with a cocktail of biotinylated detection antibodies. The sample/antibody mixture was then incubated with the array membrane overnight at 4 °C. The membranes were washed and incubated with streptavidin–horseradish peroxidase followed by chemiluminescent detection. The array data were quantitated to generate a protein profile and results are presented as average signal (pixel density) of the pair of duplicate spots representing each cytokine or chemokine analyzed using Image-J software. The data presented is from three independent repeats.

### Statistical analysis

All data are presented as the mean ± S.E.M. and were analyzed statistically by ANOVA with Tukey/Sudak post-hoc analysis using GraphPad Prism. Comparisons of groups of data were performed using an unpaired two-tailed Student’s t-test, and statistical significance is shown in figures (*p < 0.05, **p < 0.01, ***p < 0.001) where specified.

## Supplementary Information

Supplementary Information 1.

Supplementary Information 2.
