# Supplementary material for: JAK/STAT inhibitor therapy partially rescues the lipodystrophic autoimmune phenotype in Clec16a KO mice
Source: Sci Rep. 2021 Apr 1;11:7372. doi: 10.1038/s41598-021-86493-8 (PMC8016875; doi:10.1038/s41598-021-86493-8)

JAK/STAT inhibitor therapy partially rescues the lipodystrophic autoimmune phenotype in *Clec16a* KO mice

Authors: Rahul Pandey[1], Marina Bakay[1], Bryan P. Strenkowski[1], Heather S. Hain[1], and Hakon Hakonarson[1, 2]*

Affiliations:
[1]The Center for Applied Genomics, The Children's Hospital of Philadelphia, Philadelphia, PA, USA 19104.

[2]Department of Pediatrics, The Perelman School of Medicine, University of Pennsylvania, Philadelphia, PA, USA 19104.
* E-mail: hakonarson@email.chop.edu

Supplementary Figure 1.

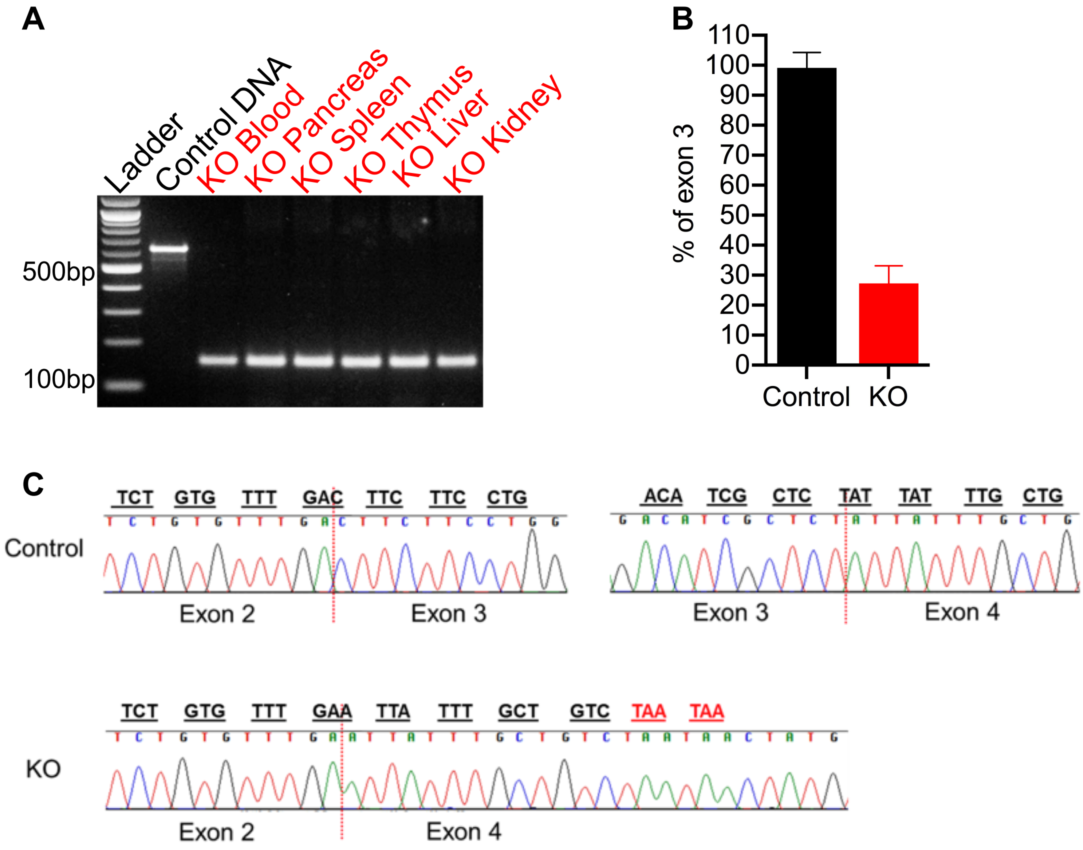

Supplementary Figure 1. Confirmation of exon-3 excision in *Clec16a*$^{\Delta UBC}$ KO mice. (A) Regular PCR of genomic DNA was performed using Clec16a-LoxP-Forward 5'-TGTGTTGTTCTCCCTTGCAG-3';Clec16a-LoxP-Reverse5' GAATAGTGGGCAAACACACGCCACTA-3'; primers. PCR product of 618bp in control and 146bp in KO confirms removal of *CLEC16A* exon 3 in KO mice. (B) Representative RT-PCR analysis from genomic DNA depicting percent of exon 3 in control (n=19) and KO (n=25) mice using 20X TaqMan assays (Applied Biosystems) TaqMan Copy Number Reference, Tert (4458373) Mm00445474_cn Clec16a (PN4400292). After recombination KO retains 30% of exon 3 in comparison to control. Data are presented as Mean±SE. (C) Sanger sequence of cDNA from blood confirmed induced skipping of exon 3, frameshift of the reading frame and generation of STOP codons in exon 4. cDNA was amplified using Mm-Clec16a-cDNA_ex1-Forward 5'ACATCCACTCCTTGGACCAC-3' and Mm-Clec16a-cDNA_ex7-Reverse 5'-TGCTCCTCATCTGTCTGCAC-3' primers. PCR products were purified from agarose gel and sequenced using the same forward and reverse primers.

Supplementary Table 1.

Table 1. Primer sequences for real-time qPCR analysis.		
Gene	Forward primers (5’- 3’)	Reverse primers (5’- 3’)
**Genes regulating ER Stress**		
Grp78	GAGACTGCTGAGGCGTATTT	CAGCATCTTTGGTTGCTTGTC
Atf6	CGGTCCACAGACTCGTGTTC	GCTGTCGCCATATAAGGAAAGG
Ire1a	CATCGGCCTTTGCTGATAGTC	CCGGGTCTTGGTGTCATACAT
Xbp1	AGCAGCAAGTGGTGGATTTG	GAGTTTTCTCCCGTAAAAGCTGA
Chop	CTGGAAGCCTGGTATGAGGAT	CAGGGTCAAGAGTAGTGAAGGT
Actin	AGCCATGTACGTAGCCATCCA	TCTCCGGAGTCCATCACAATG
**Genes regulating lipid metabolism**		
Cpt1b	ATCATGTATCGCCGCAAACT	CCATCTGGTAGGAGCACATGG
Ppara	AGAGCCCCATCTGTCCTCTC	ACTGGTAGTCTGCAAAACCAAA
Pparg	GCATGGTGCCTTCGCTGA	TGGCATCTCTGTGTCAACCATG
Adipoq	GCACTGGCAAGTTCTACTGCAA	GTAGGTGAAGAGAACGGCCTTGT
Ucp1	CTTTGCCTCACTCAGGATTGG	ACTGCCACACCTCCAGTCATT
Cidea	GCCGTGTTAAGGAATCTGCTG	TGCTCTTCTGTATCGCCCAGT

Supplementary Figure 2.

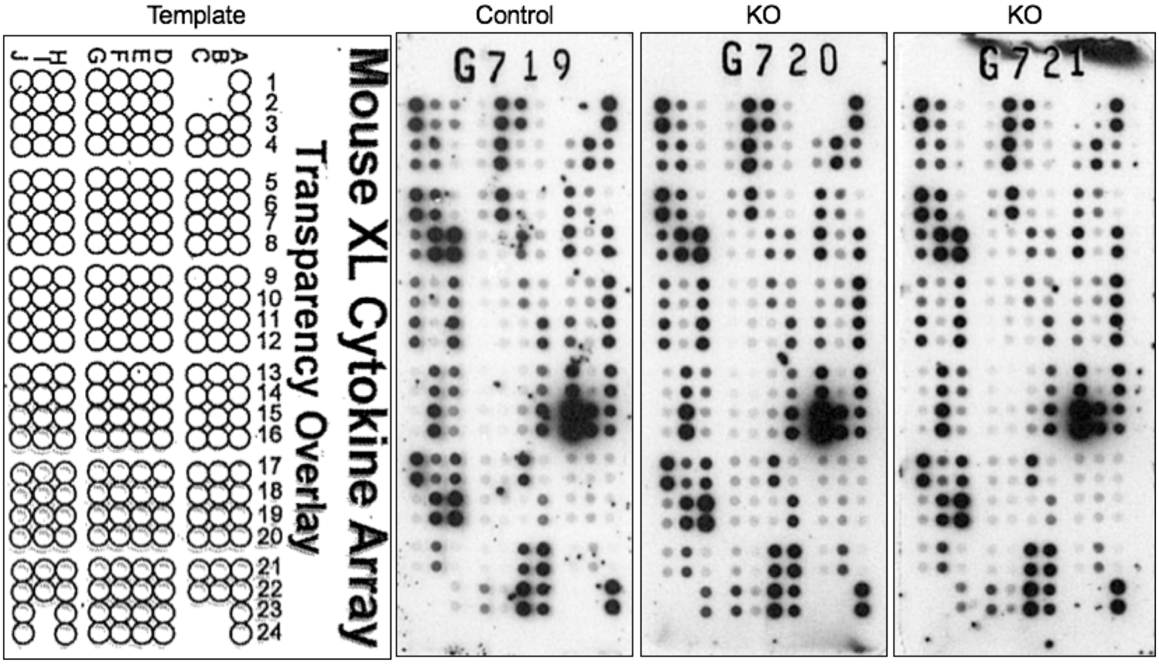

Supplementary Figure 2. Mouse XL Cytokine Array. Representative Array blot image of the plasma cytokine and chemokine from gWAT lysate of Control and KO mice (≤10% body weight loss).

Supplementary Figure 3.

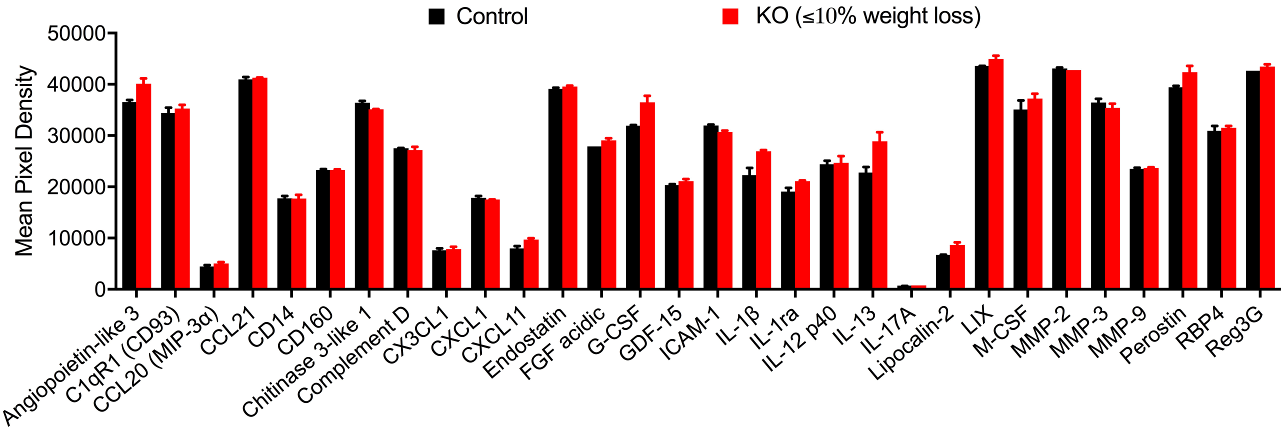

Supplementary Figure 3. Mouse XL Cytokine Array. (A) Representative Array blot image of the plasma cytokine and chemokine from gWAT lysate of Control and KO mice (≤10% body weight loss). Equal concentration of WAT lysate was run on the array. Image shown is from a two-hour exposure to X-ray film. The average signal (pixel density) of the pair of duplicate spots representing each cytokine or chemokine was analyzed using Image-J software. We evaluated the gWAT lysate of control and *Clec16a* KO mice in the Proteome Profiler Mouse XL Cytokine Array for 111 serum markers consisting of cytokines, adipokines, growth factors and other immune related proteins. We observed a potential dynamic immune response depicting significant upregulation of cytokines, chemokines and growth factor genes (fig 3 A-D). Angiopoietin-1 and -2, B-cell activating factor (BAFF), upregulation of several chemokines: CCL3, CCL5 CCL11, CC17, CD40 (Receptor for TNFSF5/CD40LG), Coagulation Factor III, CXCL2, CXCL9, CXCL10, CXCL13, CXCL16, DKK-1, CD26, EGF, Endoglin (CD105), FGF-2, IGFBP-1, IGFBP-2, IGFBP-3, IGFBP-5, Cytokines: IL-2, IL-3, IL-4, IL-5, IL-6, IL-7, Gas 6, G-CSF, GM-CSF, HGF, IFN-γ, IL-10, IL-11, IL-13, IL-15, IL-22, IL-23, IL-27 p28, IL-28A/B, IL-33, LDL R, LIF, Myeloperoxidase, Osteopontin, Osteoprotegerin, PD-ECGF, PDGF-BB, Pentraxin 2, Pentraxin 3, Pref-1, Proliferin, "Proprotein Convertase 9, RAGE, Resistin, E-Selectin, P-Selectin, Serpin F1, Serpin E1, Thrombopoietin, TIM-1, TNF-α, VCAM-1, VEGF and WISP-1. We saw significant downregulation of Adiponectin, CCL6, CCL19, IL-1α and Leptin (fig 3D). No significant change was observed for Angiopoietin-like 3, C1qR1 (CD93), CCL20 (MIP-3α), CCL21, CD14, CD160, Chitinase 3-like 1, Complement D, CX3CL1, CXCL1, CXCL11, Endostatin, FGF acidic, G-CSF, GDF-15, ICAM-1, IL-1β, IL-1ra, IL-12 p40, IL-13, IL-17A, Lipocalin-2, LIX, M-CSF, MatrixMMP-2, MMP-3, MMP-9, Perostin, RBP4 and Reg3G (Sup Fig 3).

Supplementary Table 2. Table depicting Mouse XL Cytokine Array coordinates.

Coordinate	Analyte/Control
A1, A2	Reference Spot
A3, A4	Adiponectin/Acrp30
A5, A6	Amphiregulin
A7, A8	Angiopietin-1
A9, A10	Angiopietin-2
A11, A12	Angiopietin-like 3
A13, A14	BAFF/BLyS/TNFSF13B
A15, A16	C1qR1/CD93
A17, A18	CCL2/JE/MCP-1
A19, A20	CCL3/CCL4/MIP-1α/β
A21, A22	CCL5/RANTES
A23, A24	Reference Spot
B3, B4	CCL6/C10
B5, B6	CCL11/Eotaxin
B7, B8	CC12/MCP-5
B9, B10	CC17/TARC
B11, B12	CCL19/MIP-3β
B13, B14	CCL20/MIP-3α
B15, B16	CCL21/6Ckine
B17, B18	CCL22/MDC
B19, B20	CD14
B21, B22	CD40/TNFRSFS
C3, C4	CD160
C5, C6	Chemerin
C7, C8	Chitinase 3-like 1
C9, C10	Coagulation Factor II
C11, C12	Complement Component C5/C5a
C13, C14	Complement D
C15, C16	C-Reactive Protein/CRP
C17, C18	CX3CL1/Fractalkine
C19, C20	CXCL1/KC
C21, C22	CXCL2/MIP-2
D1, D2	CXCL9/MIG
D3, D4	CXCL10-IP-10
D5, D6	CXCL11/I-TAC
D7, D8	CXCL13/BLC/BCA-1
D9, D10	CXCL16
D11, D12	Cystatin C
D13, D14	DKK-1
D15, D16	DPPIV/CD26
D17, D18	EGF
D19, D20	Endoglin/CD105
D21, D22	Endostatin
D23, D24	Fetuin
E1, E2	FGF acidic
E3, E4	FGF-21
E5, E6	Fit-3 Ligand
E7, E8	Gas 6
E9, E10	G-CSF
E11, E12	GDF-15
E13, E14	GM-CSF
E15, E16	HGF
E17, E18	ICAM-1/CD54
E19, E20	IFN-γ
E21, E22	IGFBP-1
E22,24	IGFBP-2
F1, F2	IGFBP-3
F3, F4	IGFBP-5
F5, F6	IGFBP-6
F7, F8	IL-1α/IL-1F1
F9, F10	IL-1β/IL-1F2
F11, F12	IL-1ra/IL-1F3
F13, F14	IL-2
F15, F16	IL-3
F17, F18	IL-4
F19, F20	IL-5
F21, F22	IL-6
F23, F24	IL-7
G1, G2	IL-10
G3, G4	IL-11
G5, G6	IL-12 p40
G7, G8	IL-13
G9, G10	IL-15
G11, G12	IL-17A
G13, G14	IL-22
G15, G16	IL-23
G17, G18	IL-27 p28
G19, G20	IL-28A/B
G21, G22	IL-33
G23, G24	LDL R
H1, H2	Leptin
H3, H4	LIF
H5, H6	Lipocalin-2/NGAL
H7, H8	LIX
H9, H10	M-CSF
H11, H12	MMP-2
H13, H14	MMP-3
H15, H16	MMP-9
H17, H18	Myeloperoxidase
H19, H20	Osteopontin (OPN)
H21, H22	Osteoprotegerin/TNFRSF11B
H23, H24	Thymidine phosphorylase (PD-EGF)
I1, I2	PDGF-BB
I3, I4	Pentraxin 2/SAP
I5, I6	Pentraxin 3/TSG-14
I7, I8	Perostin/OSF-2
I9, I10	Pref-1/DLK-1/FA1
I11, I12	Proliferin
I13, I14	Proprotein Convertase 9/PCSK9
I15, I16	RAGE
I17, I18	RBP4
I19, I20	Reg3G
I21, I22	Resistin
J1, J2	Reference Spots
J3, J4	E-Selectin/CD62E
J5, J6	P-Selectin/CD62P
J7, J8	Serpin F1/PEDF
J9, J10	Serpin E1/PAI-2
J11, J12	Thrombopoietin
J13, J14	TIM-1/KIM-1/HAVCR
J15, J16	TNFα
J17, J18	VCAM-1/CD106
J19, J20	VEGF
J21, J22	WISP-1/CCN4
J23, J24	Negative Control

Supplementary Figure 4.

A. The *CIITA-DEXI-CLEC16A-SOCS1* gene complex on 16p13

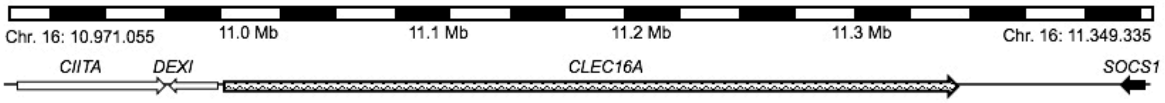

B. The *CLEC16A* gene

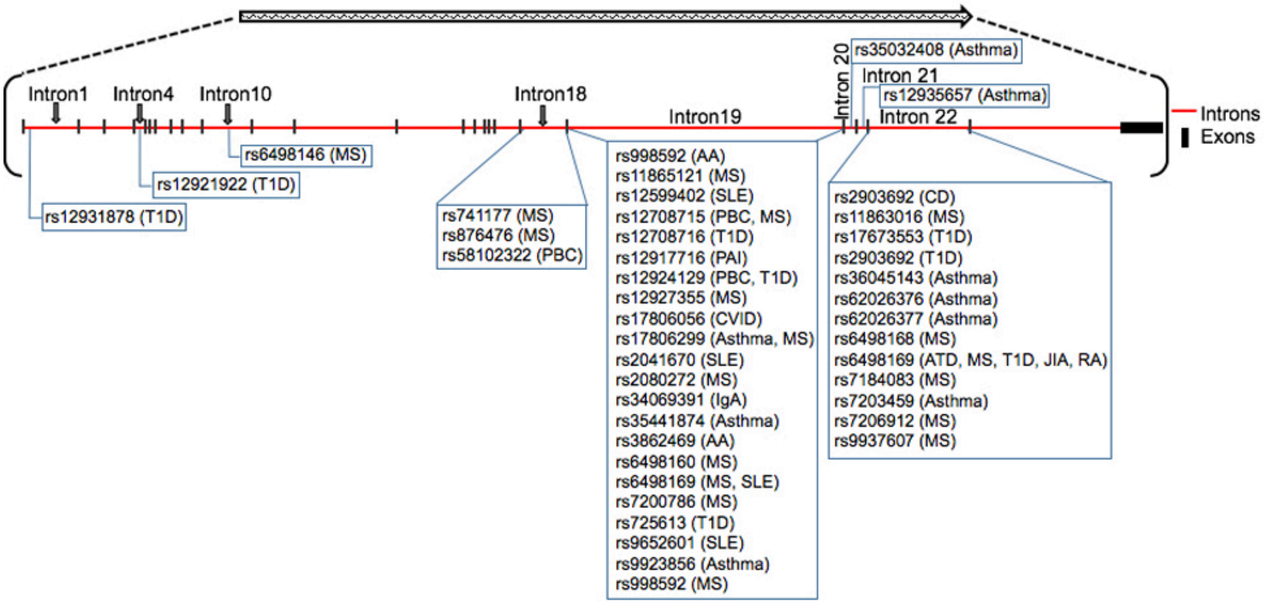

Supplementary Figure 4. (A) Schematic drawing of the chromosome 16p13 genetic region comprising *CIITA, DEXI, CLEC16A* and *SOCS1* (Genome Reference Consortium Human Build 38 (GRCh38), chromosome 16: 10.971.055–11.349.335). (B) The 238kb *CLEC16A* gene (GRCh38, chromosome 16: 11.038.345–11.276.046), where the autoimmunity-associated single nucleotide polymorphisms (SNPs) and their localization are depicted.

Supplementary Figure 5.

A

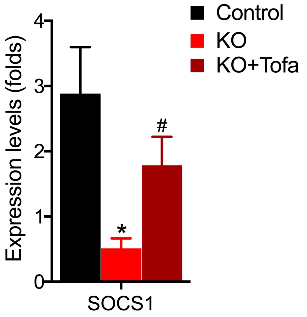

B

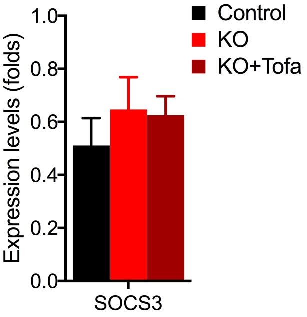

C

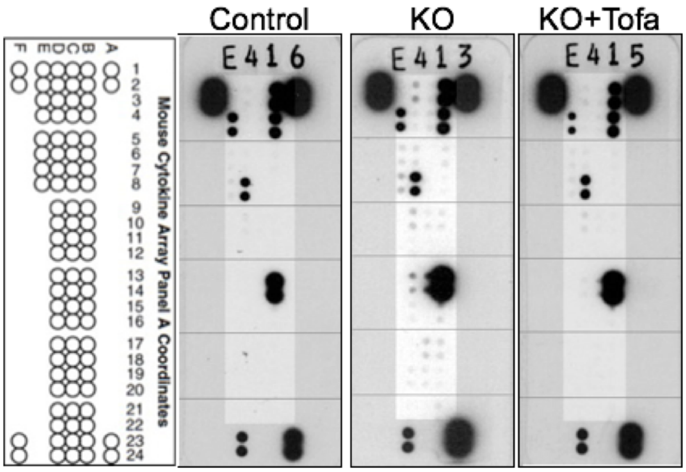

Supplementary Figure 5. Mouse plasma Cytokine Array. RT-PCR depicting SOCS1 (A) and SOCS3 (B) fold change expression in gWAT isolated from control, KO and Tofacitinib treated mice. (C) Representative Array blot of the plasma cytokine and chemokine from Control, KO and KO+Tofacitinib mice. 100 ul of plasma was run on the array. Image shown is from a two-hour exposure to X-ray film. The average signal (pixel density) of the pair of duplicate spots representing each cytokine or chemokine was analyzed using Image-J software. (D) Table depicts cytokines, chemokines, adipokines, growth factors and immune related proteins coordinates on Mouse Cytokine Array Panel A.

D

**Coordinate**	**Target/Control**
A1, A2	Reference Spot
A23, A24	Reference Spot
B1, B2	BLC (CXCL13/BCA-1)
B3, B4	C5/C5a
B5, B6	G-CSF
B7, B8	GM-CSF
B9, B10	I-309 (CCL1/TCA-3)
B11, B12	Eotaxin (CCL11)
B13, B14	sICAM-1 (CD54)
B15, B16	IFN-γ
B17, B18	IL-1α
B19, B20	IL-1β
B21, B22	IL-1ra
B23, B24	IL-2
C1, C2	IL-3
C3, C4	IL-4
C5, C6	IL-5
C7, C8	IL-6
C9, C10	IL-7
C11, C12	IL-10
C13, C14	IL-13
C15, C16	IL-12p70
C17, C18	IL-16
C19, C20	IL-17
C21, C22	IL-23
C23, C24	IL-27
D1, D2	IP-10 (CXCL10/CRG-2)
D3, D4	I-TAC(CXCL11)
D5, D6	KC (CXCL1)
D7, D8	M-CSF
D9, D10	JE (CCL2/MCP-1)
D11, D12	MCP-5(CCL12)
D13, D14	MIG (CXCL9)
D15, D16	MIP-1α (CCL3)
D17, D18	MIP-1β (CCL4)
D19, D20	MIP-2 (CXCL2)
D21, D22	RANTES (CCL5)
D23, D24	SDF-1 (CXCL12)
E1, E2	TARC
E3, E4	TIMP-1
E5, E6	TNFα
E7, E8	TREM-1
F1, F2	Reference Spot
F23, F24	PBS (negative Control)

Figure 1E. Full Blots

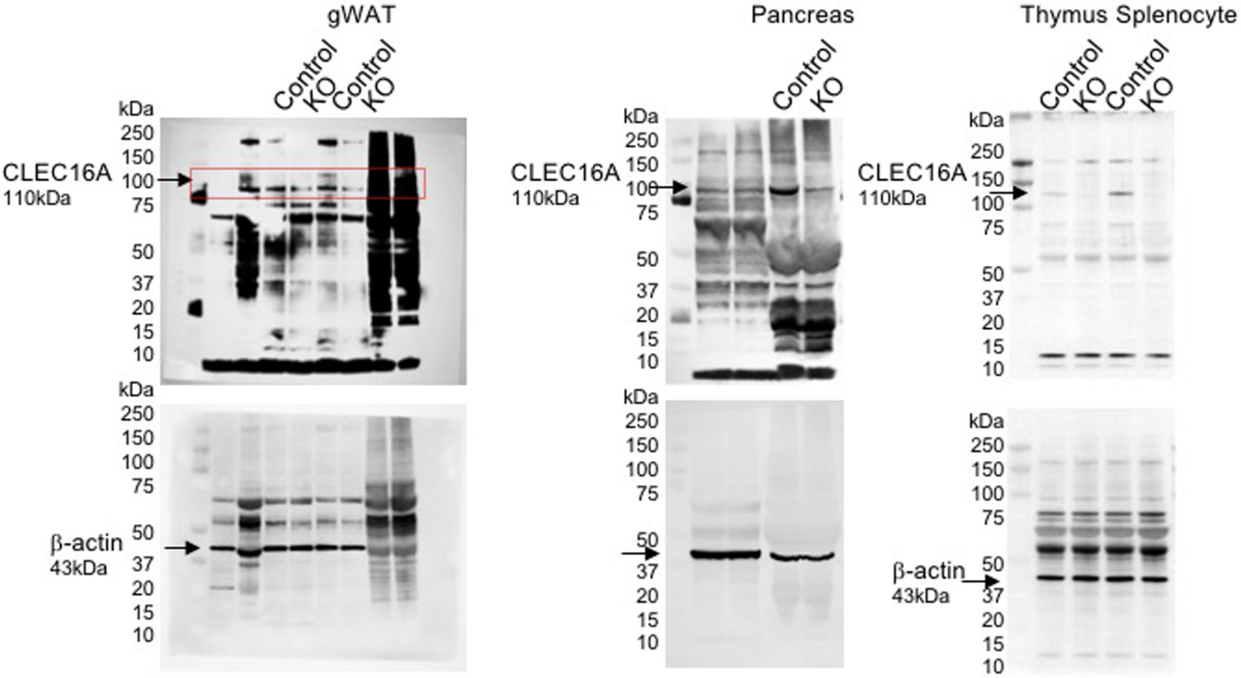

Figure 1H. Full blots

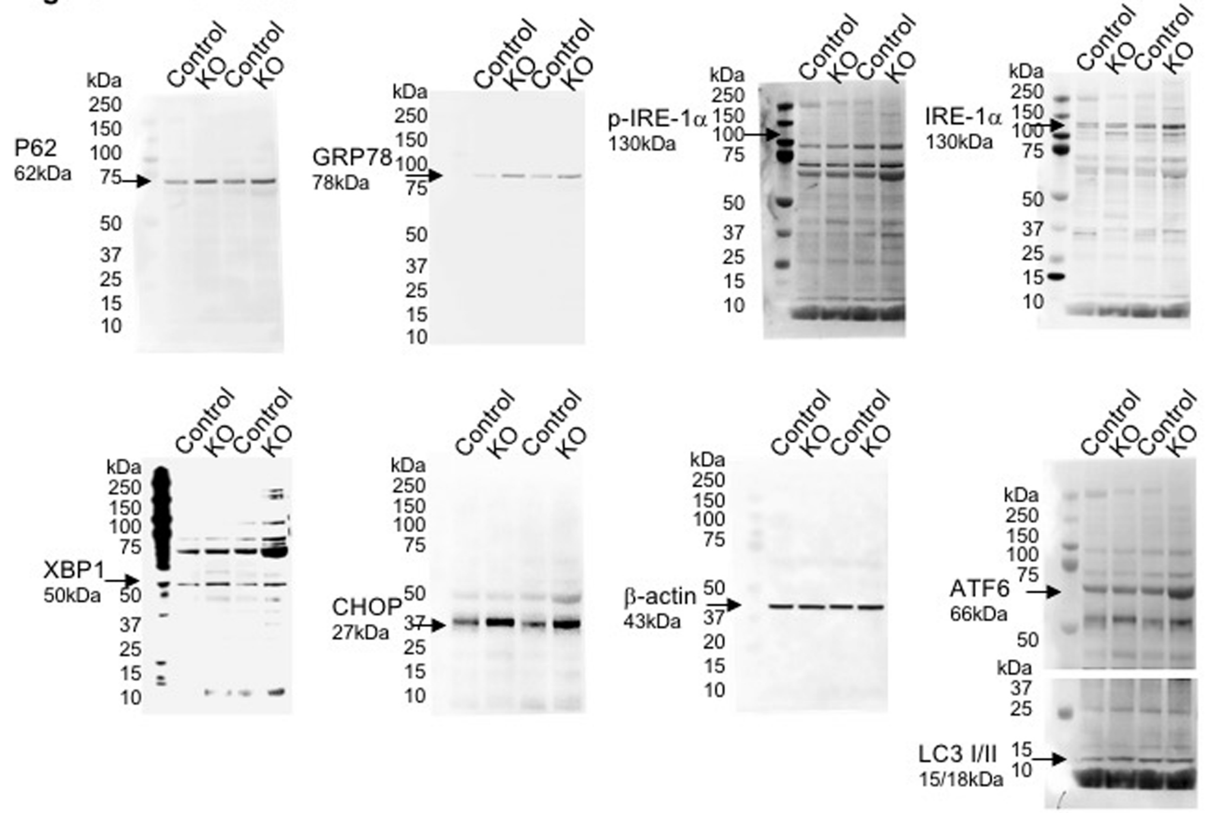

Figure 2A. Full Blots

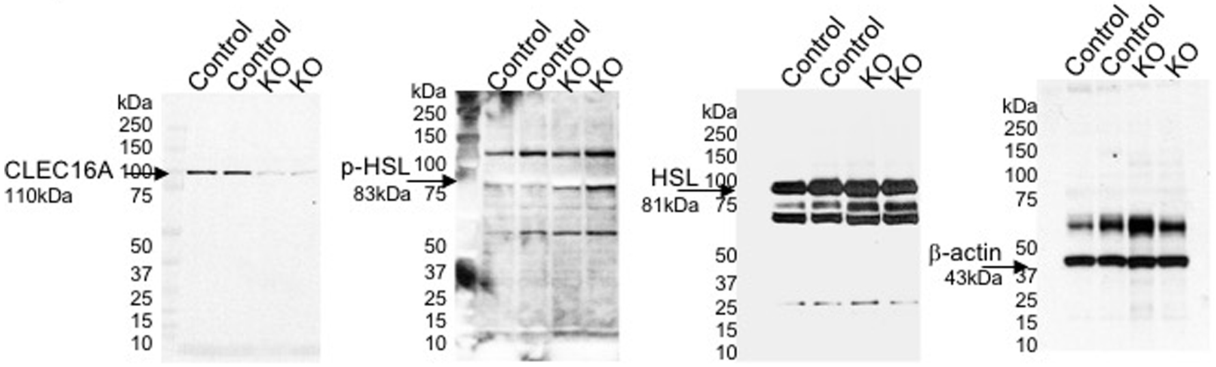

Figure 2D.

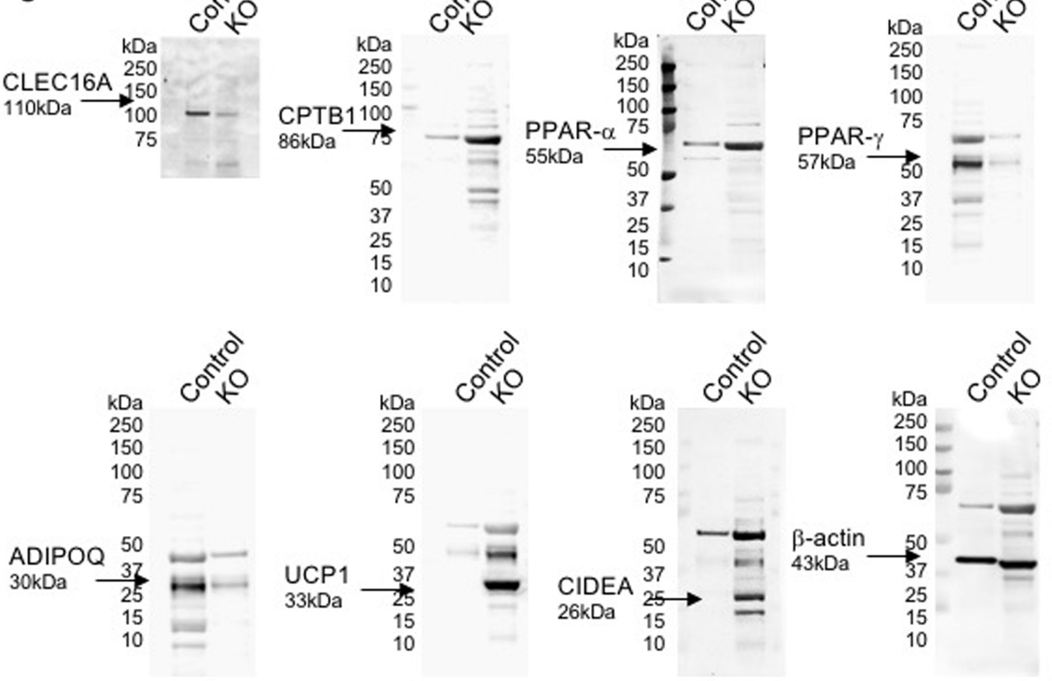

Figure 3E. Full Blots

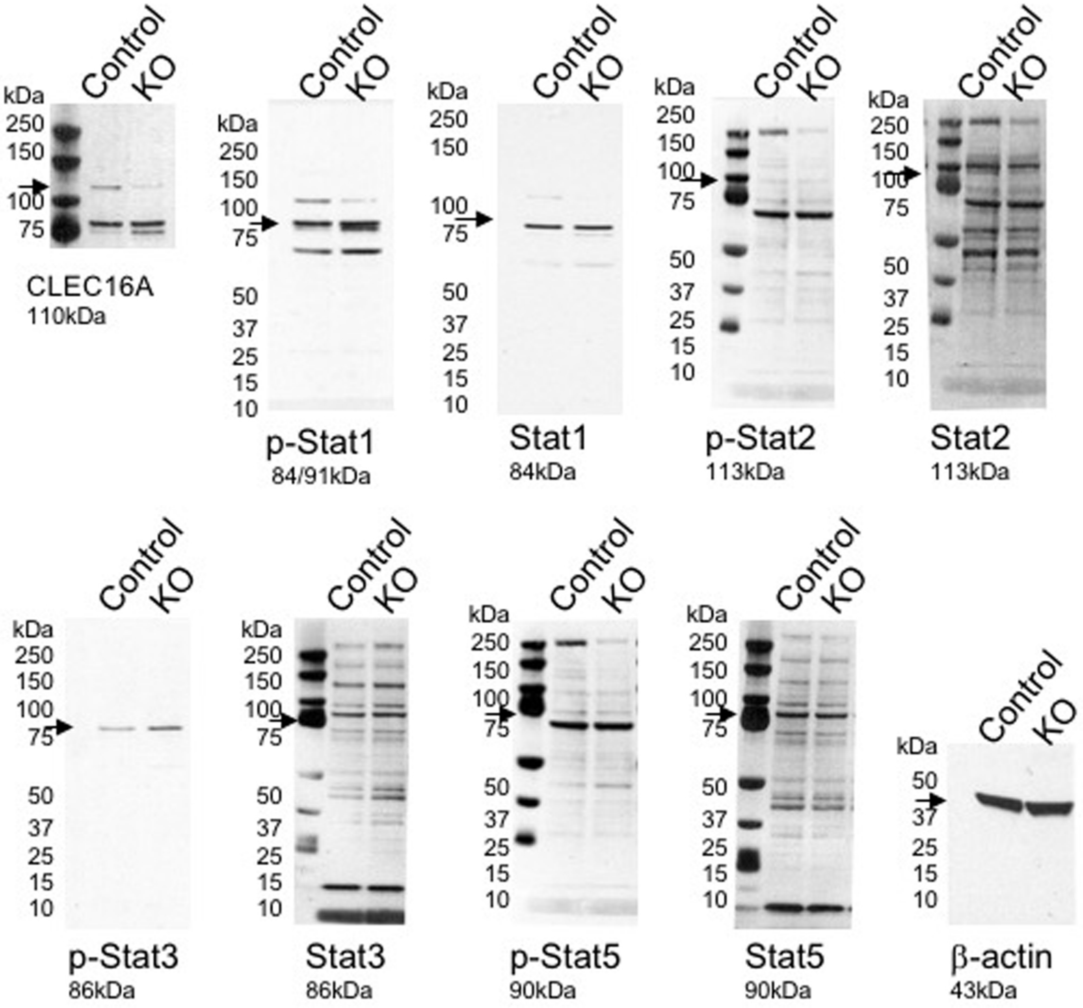

Figure 3M. Full Blots

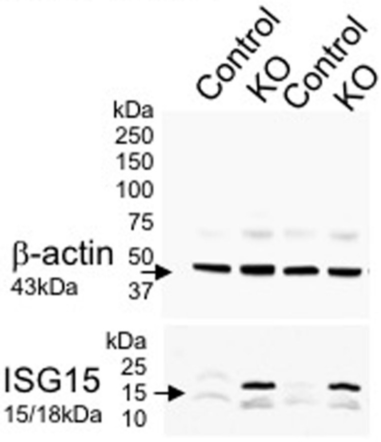

Figure 3G. Full Blots

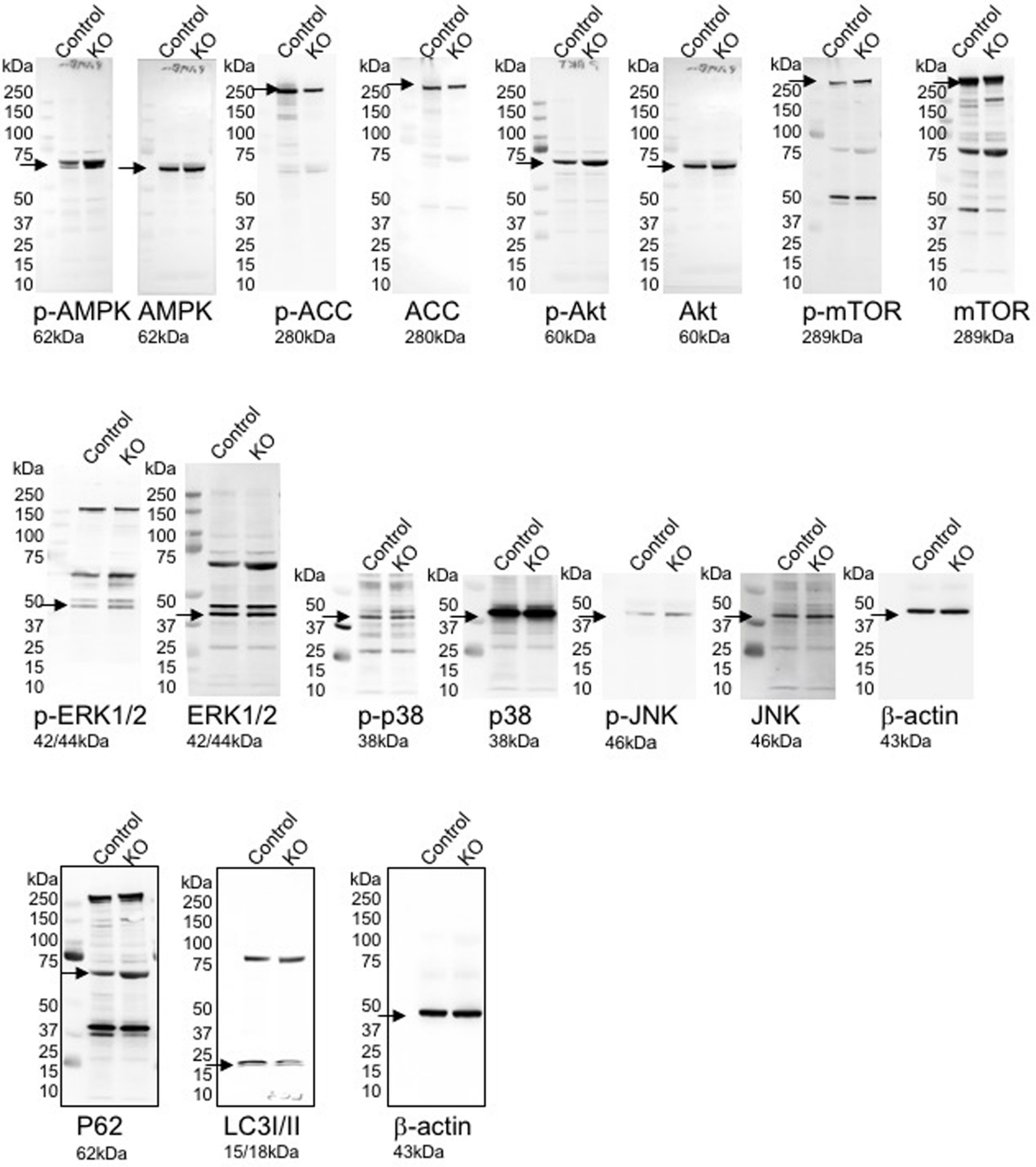

Figure 4C. Full Blots

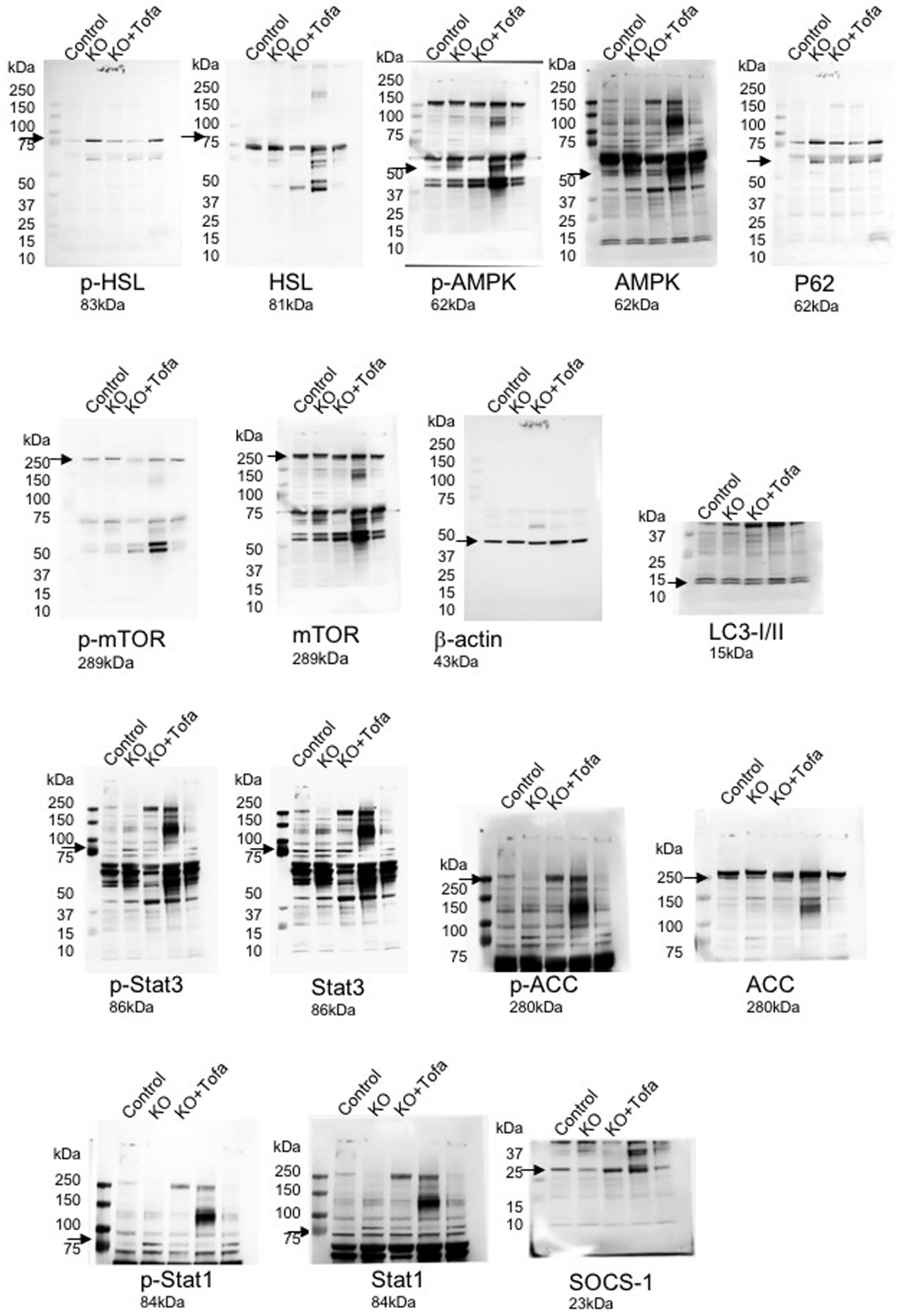

Figure 4E. Full Blots

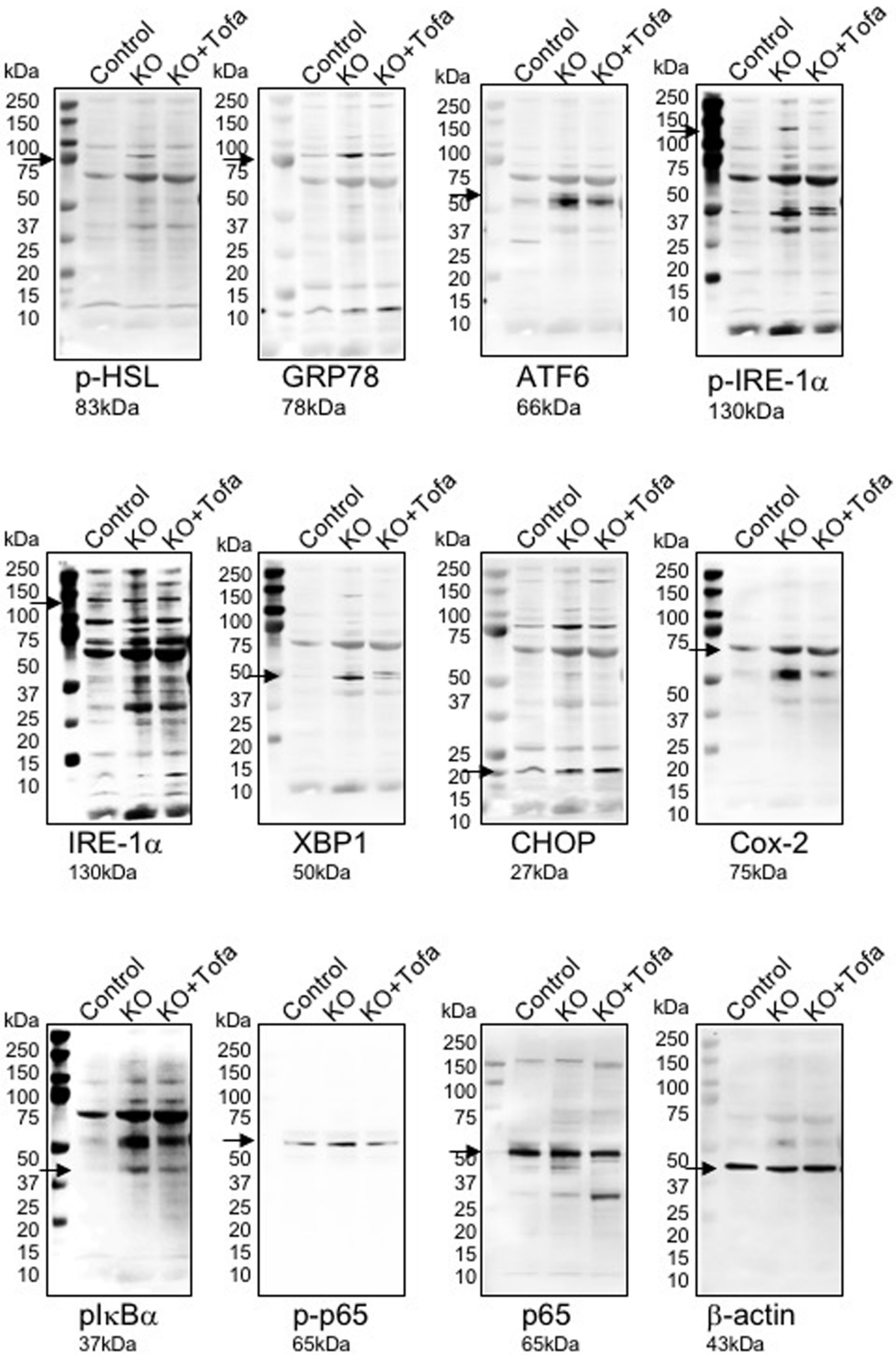

Supplement: Supplementary file 1 — Supplementary Information 1. [file 41598_2021_86493_MOESM1_ESM.pdf]
