# Supplementary material for: JAK/STAT inhibitor therapy partially rescues the lipodystrophic autoimmune phenotype in Clec16a KO mice
Source: Sci Rep. 2021 Apr 1;11:7372. doi: 10.1038/s41598-021-86493-8 (PMC8016875; doi:10.1038/s41598-021-86493-8)
Supplement: Supplementary file 2 — Supplementary Information 2. [file 41598_2021_86493_MOESM2_ESM.docx]

**Supplementary Table 3. CLEC16A SNPs associated with autoimmune diseases.**

| **Disease** | **SNP** | **Intron** | | | | | | **Cases/Controls/Trios** | | **Subject cohort** | | **Reference** | | | **PubMed Link** |
| --- | --- | --- | --- | --- | --- | --- | --- | --- | --- | --- | --- | --- | --- | --- | --- |
| Alopecia areata | rs998592 | | 19 | | | | | 1702/1723 | European ancestry | | Jagielska, 2012 | | <https://www.ncbi.nlm.nih.gov/pubmed/22534877> | | |
|  | rs3862469 | | 19 | | | | | 2,332 / 5,233 | European ancestry | | Betz, 2015 | | [www.ncbi.nlm.nih.gov/pubmed/25608926](http://www.ncbi.nlm.nih.gov/pubmed/25608926) | | |
| Asthma | rs9923856 | | 19 | | | | | 1750/9245 | African  and Europian Americans | | Almoguera, 2017 | | [www.ncbi.nlm.nih.gov/pubmed/27611488](http://www.ncbi.nlm.nih.gov/pubmed/27611488) | | |
|  | rs62026377 | | 22 | | | | |  |  |  |  |  |  |  |  |
|  | rs7203459 | | 22 | | | | | 28,399 /128,843 | European ancestry | | Pickrell, 2016 | | [www.ncbi.nlm.nih.gov/pubmed/27182965](http://www.ncbi.nlm.nih.gov/pubmed/27182965) | | |
|  | rs17806299 | | 19 | | | | | 19,954 / 107,715 | European ancestry | | Demenais, 2018 | | www.ncbi.nlm.nih.gov/pubmed/29273806 | | |
|  | rs62026376 | | 22 | | | | | 6,685 / 14,091 | European ancestry | | Ferreira, 2014 | | www.ncbi.nlm.nih.gov/pubmed/24388013 | | |
|  | rs12935657 | | 21 | | | | | 40,544 / 300,671 | European ancestry | | Ferreira, 2019 | | [www.ncbi.nlm.nih.gov/pubmed/30929738](http://www.ncbi.nlm.nih.gov/pubmed/30929738) | | |
|  | rs35441874 | | 19 | | | | |  |  |  |  |  |  |  |  |
|  | rs35032408 | | 20 | | | | | 21,564/ 318,237 | British ancestry | | Pividori, 2019 | | www.ncbi.nlm.nih.gov/pubmed/31036433 | | |
|  | rs7203459 | | 22 | | | | | 5,135 / 25,675 | European ancestry | | Shrine, 2019 | | [www.ncbi.nlm.nih.gov/pubmed/30552067](http://www.ncbi.nlm.nih.gov/pubmed/30552067) | | |
|  | rs35441874 | | 19 | | | | | 46,802 / 347,481 | European ancestry | | Zhu, 2019 | | www.ncbi.nlm.nih.gov/pubmed/31619474 | | |
|  | rs36045143 | | 22 | | | | | 39,770/76,768 | European ancestry | | Zhu, 2018 | | [www.ncbi.nlm.nih.gov/pubmed/29785011](http://www.ncbi.nlm.nih.gov/pubmed/29785011) | | |
| Autoimmune thyroid diseases | rs6498169 | | 22 | | | | | 667/301 | Chinese Han | | Muhali, 2014 | | <https://www.ncbi.nlm.nih.gov/pubmed/24646814> | | |
| Common variable immunodeficiency | rs17806056 | | 19 | | | | | 778 / 10,999 | Sweden, Norway,  USA, UK, Germany | | Li, 2015 | | <https://www.ncbi.nlm.nih.gov/pubmed/25891430> | | |
| Crohn’s disease | rs2903692 | | 22 | | | | | 1264/890 | Spain | | Marquez, 2009 | | <https://www.ncbi.nlm.nih.gov/pubmed/19337309> | | |
| Juvenile idiopathic arthritis (JIA) | rs6498169 | | 22 | | | | | 1318/2149 | Norway | | Skinningsrud, 2010 | | <https://www.ncbi.nlm.nih.gov/pubmed/19734133> | | |
| Multiple sclerosis | rs6498169 | | 22 | | | | | 2322/5418/1540 | European ancestry | | Hafler, 2007 | | [www.ncbi.nlm.nih.gov/pubmed/17660530](http://www.ncbi.nlm.nih.gov/pubmed/17660530) | | |
|  | rs11865121 | | 19 | | | | | 2,624 / 7,220 | European ancestry | | De Jager, 2009 | | www.ncbi.nlm.nih.gov/pubmed/19525953 | | |
|  | rs7200786 | | 19 | | | | | 9,772 /16,849 | European ancestry | | Sawcer, 2011 | | www.ncbi.nlm.nih.gov/pubmed/21833088 | | |
|  | rs12927355 | | 19 | | | | | 14,498 / 24,091 | European ancestry | | Beecham, 2013 | | www.ncbi.nlm.nih.gov/pubmed/24076602 | | |
|  | rs4780346 | | intragenic region | | | | |  |  |  |  |  |  |  |  |
|  | rs6498168 | | 22 | | | | | 4,888 / 10,395 | German ancestry | | Andlauer, 2016 | | [www.ncbi.nlm.nih.gov/pubmed/27386562](http://www.ncbi.nlm.nih.gov/pubmed/27386562) | | |
|  | rs6498160 | | 19 | | | | | 2,273/ 2,148 | Sardinian | | Steri, 2017 | | www.ncbi.nlm.nih.gov/pubmed/28445677 | | |
|  | rs6498169 | | 22 | | | | | 1146/1309 | Australia | | Rubio, 2008 | | <https://www.ncbi.nlm.nih.gov/pubmed/18650830> | | |
|  | rs725613 | | 19 | | | | | 1498/1706 | Sardinia | | Zoledziewska, 2009 | | <https://www.ncbi.nlm.nih.gov/pubmed/18946483> | | |
|  | rs12708716 | | 19 | | | | | 5737/10296/2369 | Australia, Belgium,  Norway, Sweden, UK, USA | | International Multiple Sclerosis Genetics Consortium, 2009 | | <https://www.ncbi.nlm.nih.gov/pubmed/18987646> | | |
|  | rs6498169 | | 19 | | | | | 1146/1309 | Europe | | Perera, 2009 | | <https://www.ncbi.nlm.nih.gov/pubmed/19375175> | | |
|  | rs6498146 | | 10 | | | | |  |  |  |  |  |  |  |  |
|  | rs741177 | | 18 | | | | |  |  |  |  |  |  |  |  |
|  | rs876476 | | 18 | | | | |  |  |  |  |  |  |  |  |
|  | rs11863016 | | 22 | | | | |  |  |  |  |  |  |  |  |
|  | rs9937607 | | 22 | | | |  | |  |  |  |  |  |  |  |
|  | rs11865121 | | 19 | | | | 2624/7220 | | UK, USA | | De Jager, 2009 | | www.ncbi.nlm.nih.gov/pubmed/19525953 | | |
|  | rs6498169 | | 22 | | | | 435/550 | | Spain | | Martinez, 2010 | | https://www.ncbi.nlm.nih.gov/pubmed/19221398 | | |
|  | rs6498169 | | 22 | | | | 211/182 (+521 multiplex controls) | | UK | | D’Netto, 2009 | | <https://www.ncbi.nlm.nih.gov/pubmed/19506219> | | |
|  | rs6498169 | | 22 | | | | 1853/2128 | | Holland, Can. | | Hoppenbrouwers, 2009 | | <https://www.ncbi.nlm.nih.gov/pubmed/19834503> | | |
|  | rs12708716 | | 19 | | | | 918/656 | | African Americans | | Johnson, 2010 | | <https://www.ncbi.nlm.nih.gov/pubmed/19865102> | | |
|  | rs6498169 | | 22 | | | |  |  |  |  |  |  |  |  |  |
|  | rs2080272 | | 19 | | | |  |  |  |  |  |  |  |  |  |
|  | rs2041670 | | 19 | | | | 603/825 | | Europe | | Nischwitz, 2011 | | <https://www.ncbi.nlm.nih.gov/pubmed/20849399> | | |
|  | rs998592 | | 19 | | | | 197/197 | | India | | Pandit, 2011 | | <https://www.ncbi.nlm.nih.gov/pubmed/20952449> | | |
|  | rs12708716 | | | 19 | | |  | |  |  |  |  | https://www.ncbi.nlm.nih.gov/pubmed/ | | |
|  | rs12708716 | | | 19 | | | 3102/5047/1113 | | Norway, UK | | Mero, 2011 | | <https://www.ncbi.nlm.nih.gov/pubmed/21179112> | | |
|  | rs7206912 | | | 22 | | |  |  |  |  |  |  |  |  |  |
|  | rs6498169 | | | 22 | | |  |  |  |  |  |  |  |  |  |
|  | rs7184083 | | | 22 | | | 1343/1379 | | UK, US | | Zuvich, 2011 | | [https://www.ncbi.nlm.nih.gov/pubmed/ 21653641](https://www.ncbi.nlm.nih.gov/pubmed/%2021653641) | | |
| Primary adrenal insufficiency | rs12917716 | | 19 | | | | | 542/1220 | Norway, UK | | Skinningsrud, 2008 | | <https://www.ncbi.nlm.nih.gov/pubmed/18593762> | | |
| Primary biliary cirrhosis (PBC) | rs12924729 | | 19 | | | | | 2,764 / 10,475 | European ancestry | | Cordell, 2015 | | [www.ncbi.nlm.nih.gov/pubmed/26394269](http://www.ncbi.nlm.nih.gov/pubmed/26394269) | | |
|  | rs12708715 | | 19 | | | | | 2,861 / 8,514 | British  and Irish ancestry | | Liu, 2012 | | [www.ncbi.nlm.nih.gov/pubmed/22961000](http://www.ncbi.nlm.nih.gov/pubmed/22961000) | | |
|  | rs12708715 | | 19 | | | | |  |  |  |  |  |  |  |  |
|  | rs58102322 | | 18 | | | | | 1450/2967 | Europe | | Hirschfield, 2012 | | <https://www.ncbi.nlm.nih.gov/pubmed/22257840> | | |
|  | rs12924129 | | 19 | | | | |  |  |  |  |  |  |  |  |
|  | rs12924729 | | 19 | | | | | 2460/7677 | UK | | Mells, 2011 | | www.ncbi.nlm.nih.gov/pubmed/21399635 | | |
| Rheumatoid arthritis | rs6498169 | | 22 | | | | | 600/550 | Spain | | Martinez, 2010 | | <https://www.ncbi.nlm.nih.gov/pubmed/19221398> | | |
|  | rs6498169 | | 22 | | | | | 1318/2149 | Norway | | Skinningsrud, 2010 | | <https://www.ncbi.nlm.nih.gov/pubmed/19734133> | | |
| Selective IgA deficiency | rs34069391 | | 19 | | | | | 1,635 / 4,852 | European ancestry | | Bronson, 2016 | | [www.ncbi.nlm.nih.gov/pubmed/27723758](http://www.ncbi.nlm.nih.gov/pubmed/27723758) | | |
| Systemic lupus erythematosus | rs12599402 | | 19 | | | | | 1,656 /3,394/ | Han Chinese | | Yang, 2013 | | [www.ncbi.nlm.nih.gov/pubmed/23273568](http://www.ncbi.nlm.nih.gov/pubmed/23273568) | | |
|  | rs7200786 | | 19 | | | | | 5,201 / 9,066 | European ancestry | | Bentham, 2015 | | www.ncbi.nlm.nih.gov/pubmed/26502338 | | |
|  | rs9652601 | | 19 | | | | |  |  |  |  |  |  |  |  |
|  | rs9652601 | | 19 | | | | | 5,695/10,352 | Chinese  and Europian ancestry | | Morris, 2016 | | [www.ncbi.nlm.nih.gov/pubmed/27399966](http://www.ncbi.nlm.nih.gov/pubmed/27399966) | | |
|  | rs9652601 | | 19 | | | | | 6,748 / 11,516 | European ancestry | | Langefeld, 2017 | | [www.ncbi.nlm.nih.gov/pubmed/28714469](http://www.ncbi.nlm.nih.gov/pubmed/28714469) | | |
|  | rs2041670 | | 19 | | | | |  |  |  |  |  |  |  |  |
|  | rs8054198 | | UTR | | | | |  |  |  |  |  |  |  |  |
| Type 1 diabetes | rs12708716 | | | | 19 | 2,000 / 3,000 | | | European ancestry | | Todd, 2007 | | | [www.ncbi.nlm.nih.gov/pubmed/17554260](http://www.ncbi.nlm.nih.gov/pubmed/17554260) | |
|  | rs12708716 | | | | 19 | 1,963 / 2,938 | | | European ancestry | | Wellcome Trust Case Control Consortium, 2007 | | | [www.ncbi.nlm.nih.gov/pubmed/17554300](http://www.ncbi.nlm.nih.gov/pubmed/17554300) | |
|  | rs725613 | | | | 19 | 1896/1146/873 | | | European ancestry | | Hakonarson, 2007 | | | [www.ncbi.nlm.nih.gov/pubmed/17632545](http://www.ncbi.nlm.nih.gov/pubmed/17632545) | |
|  | rs2903692 | | | | 22 |  |  |  |  |  |  |  |  |  |  |
|  | rs17673553 | | | | 22 |  |  |  |  |  |  |  |  |  |  |
|  | rs12708716 | | | | 19 | 3,561 / 4,646 / | | | European ancestry | | Cooper, 2008 | | | [www.ncbi.nlm.nih.gov/pubmed/18978792](http://www.ncbi.nlm.nih.gov/pubmed/18978792) | |
|  | rs12708716 | | | | 19 | 7,514 / 9,045 | | | European ancestry | | Barrett, 2009 | | | [www.ncbi.nlm.nih.gov/pubmed/19430480](http://www.ncbi.nlm.nih.gov/pubmed/19430480) | |
|  | rs12708716 | | | | 19 | 8,506 / 10,596 / | | | European ancestry | | Plagnol, 2011 | | | [www.ncbi.nlm.nih.gov/pubmed/21829393](http://www.ncbi.nlm.nih.gov/pubmed/21829393) | |
|  | rs12927355 | | | | 19 | 6,683 / 12,173/69 and 2,601 affected sibling pair families | | | European ancestry | | Onengut-Gumuscu, 2015 | | | [www.ncbi.nlm.nih.gov/pubmed/25751624](http://www.ncbi.nlm.nih.gov/pubmed/25751624) | |
|  | rs725613 | | | | 19 | 1037/1706 | | | Italy (Sardinia) | | Zoledziewska, 2009 | | | <https://www.ncbi.nlm.nih.gov/pubmed/18946483> | |
|  | rs2903692 | | | | 22 | 735/621 | | | Japan | | Awata, 2009 | | | <https://www.ncbi.nlm.nih.gov/pubmed/18940880> | |
|  | rs725613 | | | | 19 | 205/422 | | | China (Han) | | Wu, 2009 | | | <https://www.ncbi.nlm.nih.gov/pubmed/19178520> | |
|  | rs6498169 | | | | 22 | 316/550 | | | Spain | | Martinez, 2010 | | | 19221398 https://www.ncbi.nlm.nih.gov/pubmed/ | |
|  | rs12921922 | | | | 4 | 131/121 | | | China | | Sang, 2012 | | | [https://www.ncbi.nlm.nih.gov/pubmed/ 22778732](https://www.ncbi.nlm.nih.gov/pubmed/%2022778732) | |
|  | rs12931878 | | | | 1 |  |  |  |  |  |  |  |  |  |  |
|  | rs12708716 | | | | 19 | 1212/2513 | | | Germany | | Howson, 2011 | | | [https://www.ncbi.nlm.nih.gov/pubmed/ 21873553](https://www.ncbi.nlm.nih.gov/pubmed/%2021873553) | |
|  | rs2903692 | | | | 22 | 1743/790 | | | Japan | | Yamashita, 2011 | | | [https://www.ncbi.nlm.nih.gov/pubmed/ 22069271](https://www.ncbi.nlm.nih.gov/pubmed/%2022069271) | |

Supplementary Table 3.
